# Electroconvulsive therapy-induced volumetric brain changes converge on a common causal circuit in depression

**DOI:** 10.1038/s41380-023-02318-2

**Published:** 2023-11-20

**Authors:** Miklos Argyelan, Zhi-De Deng, Olga Therese Ousdal, Leif Oltedal, Brian Angulo, Mate Baradits, Andrew J. Spitzberg, Ute Kessler, Alexander Sartorius, Annemiek Dols, Katherine L. Narr, Randall Espinoza, Jeroen A. van Waarde, Indira Tendolkar, Philip van Eijndhoven, Guido A. van Wingen, Akihiro Takamiya, Taishiro Kishimoto, Martin B. Jorgensen, Anders Jorgensen, Olaf B. Paulson, Antoine Yrondi, Patrice Péran, Carles Soriano-Mas, Narcis Cardoner, Marta Cano, Linda van Diermen, Didier Schrijvers, Jean-Baptiste Belge, Louise Emsell, Filip Bouckaert, Mathieu Vandenbulcke, Maximilian Kiebs, René Hurlemann, Peter CR. Mulders, Ronny Redlich, Udo Dannlowski, Erhan Kavakbasi, Michael D. Kritzer, Kristen K. Ellard, Joan A. Camprodon, Georgios Petrides, Anil K. Malhotra, Christopher C. Abbott

**Affiliations:** 1 Feinstein Institutes for Medical Research, Institute of Behavioral Science, Manhasset, NY USA; 2 The Zucker Hillside Hospital, Glen Oaks, NY USA; 3 Noninvasive Neuromodulation Unit, Experimental Therapeutics and Pathophysiology Branch, National Institute of Mental Health, National Institutes of Health, Bethesda, MD USA; 4 Department of Psychiatry and Behavioral Sciences, Duke University School of Medicine, Durham, NC USA; 5 Department of Biomedicine, Faculty of Medicine, University of Bergen, Bergen, Norway; 6 Department of Radiology, Haukeland University Hospital, Bergen, Norway; 7 Department of Clinical Medicine, University of Bergen, Bergen, Norway; 8 Mohn Medical Imaging and Visualization Centre, Department of Radiology, Haukeland University Hospital, Bergen, Norway; 9 Department of Psychiatry and Psychotherapy, Semmelweis University, Budapest, Hungary; 10 Department of Psychiatry, Haukeland University Hospital, University of Bergen, Bergen, Hungary; 11 Department of Psychiatry and Psychotherapy, Central Institute of Mental Health (CIMH), Medical Faculty Mannheim, University of Heidelberg, Heidelberg, Germany; 12 Department of Psychiatry, UMC Utrecht Brain Center, University Medical Center Utrecht, Utrecht, The Netherlands; 13 Amsterdam UMC location Vrije Universiteit Amsterdam, Psychiatry, Neuroscience, Amsterdam, The Netherlands; 14 Department of Neurology, University of California Los Angeles, Los Angeles, CA USA; 15 Department of Psychiatry and Biobehavioral Sciences, University of California Los Angeles, Los Angeles, CA USA; 16 Rijnstate, Arnhem, the Netherlands; 17 Donders Institute for Brain, Cognition and Behavior, Department of Psychiatry, Nijmegen, the Netherlands; 18 Amsterdam UMC location University of Amsterdam, Department of Psychiatry, Amsterdam, The Netherlands; 19 Amsterdam Neuroscience, Amsterdam, The Netherlands; 20 Department of Neuropsychiatry Keio University School of Medicine, Tokyo, Japan; 21 Neuropsychiatry, Department of Neurosciences, Leuven Brain Institute, KU Leuven, Belgium; 22 Hills Joint Research Laboratory for Future Preventive Medicine and Wellness, Keio University School of Medicine, Tokyo, Japan; 23 Psychiatric Center Copenhagen and Department of Clinical Medicine, University of Copenhagen, Copenhagen, Denmark; 24 Neurobiological Research Unit Rigshospitalet and Department of Clinical Medicine, University of Copenhagen, Copenhagen, Denmark; 25 Service de Psychiatrie et Psychologie Médicale, Centre Expert Dépression Résistante, Fondation Fondamental, CHU Toulouse, ToNIC, Toulouse NeuroImaging Center, Univerité de Toulouse, Inserm, UPS, Toulouse, France; 26 ToNIC, Toulouse NeuroImaging Center, Univeristé de Toulouse, Inserm, UPS, Toulouse, France; 27 Department of Social Psychology and Quantitative Psychology, Universitat de Barcelona-UB, Barcelona, Spain; 28 Bellvitge Biomedical Research Institute-IDIBELL, Department of Psychiatry, Bellvitge University Hospital, Barcelona, Spain; 29 CIBERSAM, Carlos III Health Institute, Madrid, Spain; 30 Sant Pau Mental Health Research Group, Institut d’Investigació Biomèdica Sant Pau (IIB-Sant Pau), Hospital de la Santa Creu i Sant Pau, Barcelona, Spain; 31 Department of Psychiatry and Forensic Medicine, School of Medicine Bellaterra, Universitat Autònoma de Barcelona, Barcelona, Spain; 32 Department of Psychiatry, Collaborative Antwerp Psychiatric Research Institute (CAPRI), Faculty of Medicine and Health Sciences, University of Antwerp, Antwerp, Belgium; 33 Psychiatric Center Bethanie, Andreas Vesaliuslaan 39, 2980 Zoersel, Belgium; 34 University Psychiatric Center Duffel, Stationstraat 22, Duffel, 2570 Belgium; 35 Department of Psychiatry, Radboud University Medical Centre, P.O. Box 9101, 6500 HB Nijmegen, The Netherlands; 36 Geriatric Psychiatry, University Psychiatric Center-KU Leuven, Leuven, Belgium; 37 School of Medicine & Health Sciences University Hospital Oldenburg, Oldenburg, Germany; 38 Department of Psychiatry and Psychotherapy University Hospital Bonn, Bonn, Germany; 39 Department of Psychology, University of Halle, Halle, Germany; 40 German Center for Mental Health (DZPG), Site Jena-Magdeburg-Halle, Halle, Germany; 41 Department of Translational Psychiatry, University of Muenster, Muenster, Germany; 42 Department of Mental Health, University of Muenster, Muenster, Germany; 43 Department of Psychiatry, Massachusetts General Hospital, Harvard Medical School, Boston, MA USA; 44 Department of Psychiatry, University of New Mexico, Albuquerque, NM USA

**Keywords:** Predictive markers, Depression

## Abstract

Neurostimulation is a mainstream treatment option for major depression. Neuromodulation techniques apply repetitive magnetic or electrical stimulation to some neural target but significantly differ in their invasiveness, spatial selectivity, mechanism of action, and efficacy. Despite these differences, recent analyses of transcranial magnetic stimulation (TMS) and deep brain stimulation (DBS)-treated individuals converged on a common neural network that might have a causal role in treatment response. We set out to investigate if the neuronal underpinnings of electroconvulsive therapy (ECT) are similarly associated with this causal depression network (CDN). Our aim here is to provide a comprehensive analysis in three cohorts of patients segregated by electrode placement (*N* = 246 with right unilateral, 79 with bitemporal, and 61 with mixed) who underwent ECT. We conducted a data-driven, unsupervised multivariate neuroimaging analysis Principal Component Analysis (PCA) of the cortical and subcortical volume changes and electric field (EF) distribution to explore changes within the CDN associated with antidepressant outcomes. Despite the different treatment modalities (ECT vs TMS and DBS) and methodological approaches (structural vs functional networks), we found a highly similar pattern of change within the CDN in the three cohorts of patients (spatial similarity across 85 regions: *r* = 0.65, 0.58, 0.40, df = 83). Most importantly, the expression of this pattern correlated with clinical outcomes (*t* = −2.35, *p* = 0.019). This evidence further supports that treatment interventions converge on a CDN in depression. Optimizing modulation of this network could serve to improve the outcome of neurostimulation in depression.

## Introduction

One of the oldest and most effective forms of neurostimulation is electroconvulsive therapy (ECT) [1, 2] However, despite the last decades of ECT-neuroimaging research, its mechanism of action is not known. In a recently published article, Siddiqi et al. [3] showed that a common underlying neural network (Supplementary Fig. 1A) is associated with the clinical response of treatment resistant depression in transcranial magnetic stimulation (TMS) and deep brain stimulation (DBS). Additionally, dysfunctions within this network explain clinical symptoms in patients with stroke, multiple sclerosis and other forms of brain lesions [3, 4]. These results are not mere associations, but instead indicate that interference within this network could explain individual differences in treatment response. The main cortical areas associated with the common causal network included regions previously implicated in depression and emotion regulation, such as the subgenual cingulate cortex, dorsolateral prefrontal cortex, ventromedial prefrontal cortex, inferior frontal gyrus, frontal eye field, and intraparietal sulcus (Supplementary Fig. 1A, [3, 5]).

While ECT is not a localized form of treatment, the applied ECT-induced electric field (EF) has unique spatial distribution specific to an individual and the electrode placement [6–9]. High frequency EF stimulation has a direct neuroplastic effect on the brain [10–12] and is also associated with downstream biological effects through the induced seizure activity [13, 14]. In agreement with these preclinical findings, recent large-scale studies in the Global ECT-MRI Research Collaboration (GEMRIC) dataset [15] found robust volume increases [16, 17] in a wide range of cortical and subcortical regions, which correlated with the number of ECT sessions. Subsequent EF modeling based on the individual head MRI consistently demonstrated that the ECT-induced EF strongly correlated with volume increase [18–20]. These results verified that despite the widespread activation of the brain through seizure, the direct electrical stimulation effect of ECT is much more spatially selective and individually diverse than first assumed.

Despite these replicable and robust structural findings driven by the spatial distribution of the EF, their direct or indirect effect on clinical outcome remain unclear. Univariate analysis of the EF amplitude on clinical response was the subject of several previous investigations. However, the results were somewhat contradictory [18, 21, 22]. Similarly, the robust changes in volume did not translate into correlations with clinical response in most of the studies [16, 17] or indicated a relationship where volume increase in the dentate gyrus was associated with worse clinical outcome [23].

One caveat was the primarily univariate nature of these analyses. The brain regions are not independent of each other, and multivariate analysis could be more sensitive to detect network-wide changes [3]. Indeed, one follow-up analysis of the GEMRIC dataset in 192 individuals with supervised multivariate models could detect networks of regions where the weighted average of the changes correlated with clinical outcomes [24]. The results of this analysis showed that the linear combination of volume changes across 18 regions correlated with clinical outcomes. The loadings of the 18 regions showed some similarities with the spatial distribution of the causal map published by Siddiqi et al. (Supplementary Fig. 1B, *r* = 0.33, df = 16). Although these 18 regions comprise a limited coverage of the causal map, it raises the intriguing possibility that the ECT-induced volume changes might follow a similar spatial pattern already described with functional connectivity analysis in other treatment modalities such as TMS and DBS.

To address this question, we revisit and improve the analysis of EF-structure to clinical outcomes by doubling the sample size across three independent cohorts (total *N* = 386). We implement an unsupervised learning algorithm Principal Component Analysis (PCA) running separately on EF and structural data, and separately on different electrode placements (6 parallel PCAs). The non-supervised learning methods reduce the risk of overfitting. Any convergence in these independent but parallel multivariate analyses would strongly support the validity of our findings and indicate a common pathway in the mechanism of action of ECT. We propose that not only will a common principal component emerge, but it will also exhibit a resemblance to the causal circuit previously reported in the context of TMS and DBS efficacy [3].

## Methods

### Participants

386 ECT-treated subjects were analyzed from the GEMRIC consortium [15]. This multi-site consortium collects data in a centralized server from ECT-treated patients who underwent longitudinal neuroimaging and clinical assessment. The 386 subjects were recruited at 19 sites and their respective demographics and clinical data are in Supplementary Table 1. All contributing sites received ethics approval from their local ethics committee or institutional review board. In addition, the centralized mega-analysis was approved by the Regional Ethics Committee South-East in Norway (No. 2013/1032).

### Neuroimaging analysis

We calculated (1) volume changes and (2) EFs in 85 regions (Supplementary Fig. 2).

1. Volume changes
  The image processing methods have been detailed previously [16–18]. In brief, the sites provided longitudinal 3 T T1-weighted MRI images (at baseline and after the end of the course of ECT) with a minimal resolution of 1.3 mm in any direction. The raw DICOM images were uploaded and analyzed on a common server at the University of Bergen, Norway. To guarantee reproducibility, in addition to the common platform, the processing pipelines were implemented in a docker environment [25]. First, images were corrected for scanner-specific gradient-nonlinearity [26]. Further processing was performed with FreeSurfer version 7.1, which includes segmentation of subcortical structures [27] and automated parcellation of the cortex [28]. In addition to brainstem and bilateral cerebellum, this automated process identified 33 cortical and eight subcortical regions in each hemisphere. Altogether this resulted in 85 regions of interest (ROIs) (Supplementary Tables). Next longitudinal FreeSurfer analysis was used for unbiased, within-subject assessment of estimation of longitudinal volume change (ΔVol - %) (Supplementary Fig. 2). In more detail, we cross-sectionally processed both time points separately with the default FreeSurfer workflow and created an unbiased template from both time points for each subject. Once this template is created, parcellations and segmentation are carried out at each time point initialized with common information from the within-subject template [29]. In summary, we calculated bias-free estimation of volumetric change from 85 brain regions across the timespan of an ECT course in 386 individuals who received on average of 12.5 ± 5.4 ECT sessions.
2. EF modeling
  Our approach was detailed in one of our previous manuscripts [18], with the upgraded software of Roast 3.0 (Realistic Volumetric-Approach to Stimulate Transcranial Electric Stimulation v3.0) [6]. In short, ROAST builds a three-dimensional tetrahedral mesh model of the head based on the T1 MRI images of the brain. Then, segmentation identifies five tissue types: white and gray matter of the brain, cerebrospinal fluid, skull, and scalp, and assigns them different conductivity values: 0.126 S/m, 0.276 S/m, 1.65 S/m, 0.01 S/m, and 0.465 S/m respectively. ECT electrodes of 5 cm diameter were placed over the C2 and FT8 EEG (10–20 system) sites to model right unilateral (RUL), and over to FT8 and FT9 sited to model bitemporal (BT) electrode placements. Study sites from the GEMRIC database used either the Thymatron (Somatics, Venice, Florida) or spECTrum (MECTA Corp., Tualatin, Oregon) devices. EF was solved using the finite-element method with unit current on the electrodes and, subsequently, it was scaled to the current amplitude of the specific devices (Thymatron 900 mA, spECTrum 800 mA). We had 61 individuals who had to switch from RUL to BT electrode placement during the ECT course (mixed placement, MIX). This is a standard clinical practice in patients with inadequate clinical response with RUL stimulation. In these cases, we calculated the EF with the weighted mean according to the number of ECT sessions the individual had in each form of placements. For example, if a patient had 6 ECTs with RUL and then had 18 ECTs with BT then we calculated 0.25 × EF_RUL_ + 0.75 × EF_BT_ in each region. These procedures resulted in a voxel-wise EF distribution map in each individual. We calculated the average EF across the 85 three-dimensional ROIs at baseline in every individual based on the Freesurfer parcellations and segmentations. The voxel values with the top and lowest one percentile in each ROI were omitted during calculations to reduce boundary effects.

### Multivariate analysis

To investigate the regional volume changes and EF amplitudes in a multivariate way, we applied PCA. We conducted six consecutive PCA analyses on RUL, BT, and MIX separately for EF and structural data, respectively (variables were normalized across individuals before PCA). We separated the groups as we wanted to avoid capturing differences that were only electrode placement specific. We used Cattell’s scree test to determine the number of PCs to analyze. We found that the first 2 PCs captured most of the variance, and the subsequent PCs captured a diminishing portion of the variance (elbow criteria, Supplementary Fig. 3). We conducted posthoc analyses to evaluate (1) the correlation between PCs and clinical outcomes: ΔMADRS ~ PC1 + PC2 + age + nECT (nECT: number of ECT sessions, ΔMADRS: percent change compared to baseline (T2-T1)/T1, negative values indicate better response), and (2) the spatial similarity between loadings and the causal depression network (CDN) [3]. The CDN was extracted from the combined circuit maps as illustrated in Fig. 5 of Siddiqi et al. [3]. As our volumetric findings were based on 85 cortical and subcortical regions, we employed these specific regions in the MNI space to determine the average values of the voxel-based maps (the authors generously provided us with the voxel-based map for our use). Subsequently, these 85 regional values served as a reference in our computations to compare spatial similarity.

### Spatial similarity calculations

The spatial distribution of the principal components’ loadings can be compared with that of the CDN. While a straightforward approach might involve correlating these values, it’s important to note that traditional parametric correlation tests are not applicable in this case, as the regional values are not independent. To address this, we conducted two distinct permutation tests (Supplementary Material).

To investigate if one hemisphere was driving the results, we conducted the PCA separately on the right and left hemisphere (Supplementary Material).

### Covariates

We conducted multivariable regression analysis to estimate the effect of the calculated principal components on clinical response. This analysis included the principal components of the volume change, EF, age and number of ECT sessions as independent variables. These last two variables were included as confounders. As it is explained below, age correlated with EF and clinical response, and number of ECT sessions were also correlating with clinical response and volume change. Therefore, these variables had to be added to correct for spurious correlations.

### Justification of the confounding variables

We corrected for two variables consistently across our analyses. We would like to provide a brief justification for including these. We also provide a causal model with a corresponding directed acyclic causal graph to illustrate the reasoning (Supplementary Fig. 4).

1. Number of ECT sessions
  It was already noted in the first large scale publication of the GEMRIC consortium that the number of ECT sessions and clinical response correlated in a counterintuitive way: the larger the number of ECT sessions registered between MRI assessments, the lower the clinical response was. The explanation of this observation is that most of the participating sites in the GEMRIC consortium acquired the follow-up (post-ECT) MRI after completing the (un-) successful ECT course, in contrast to predetermined length of treatment period with a fixed number of ECT-sessions. This resulted in an earlier timepoint of post-ECT MRI assessment if there was a quick clinical response, but later when clinical improvement was delayed or absent. This is problematic because the number of ECT sessions positively correlates with the volume change during ECT (dose–response effect). Therefore, not controlling for the number of ECT sessions can easily lead to spurious correlations indicating that volume increase was associated with worse outcome, or just simply mask the otherwise real effect when volume change is beneficial. Indeed, in recent cohorts where the length of ECT course between the neuroimaging sessions were predetermined, authors found positive relationships between hippocampus volume increase and clinical response [19].
2. Age
  Our sample had a tight correlation between age and clinical response as well. This correlation is typical in ECT datasets [30–32], as the elderly patients respond to ECT significantly better. This introduces, however, another confound to every EF modeling as age negatively correlates with EF magnitude in the human brain due to structural changes such as atrophy [9]. This age and EF relationship was particularly strong in RUL placement (R Hippocampus; RUL: *r* = −0.31, *p* < 0.001, df = 244, BT: *r* = −0.17, *p* = 0.13, df = 77, MIX: *r* = −0.28, *p* = 0.03, df = 59), therefore it could mask the effect of EF on clinical response in our previous analysis [18].

The code relevant to this manuscript is available at https://github.com/argyelan/Publications/tree/master/VOLUME-CHANGE-PCA.

## Results

### EF correlates with volume changes

**Fig. 1:**
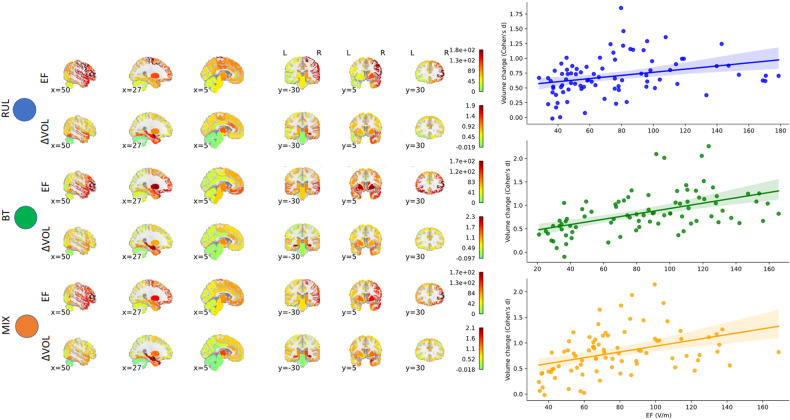
The relationship between Electric Field (EF) and volume change (ΔVOL). On the left side of the figure, brain images illustrate the spatial distribution of EF (1st, 3rd, and 5th rows, V/m) and ΔVOL (2nd, 4th, and 6th rows, Cohen’s d). The first two rows depict results from RUL placement (*n* = 246), the next two rows show results from BT placements (*n* = 79), and the last two rows display results from MIX placements (*n* = 61). These regions were segmented into 85 cortical and subcortical areas (refer to methods). Throughout the manuscript, we designated the results from RUL as blue, BT as green, and MIX as orange for color coding consistency. The right panel illustrates the corresponding values across the 85 regions, showcasing the correlation between EF and ΔVOL in RUL, BT, and MIX, respectively.

386 subjects (mean age 54 y, 233 female) with baseline MADRS 25.5 underwent an average number of 12.5 ECT sessions and experienced an average of 59% decrease in MADRS. The dataset is detailed in Supplementary Table 1A, B. Like our previous studies, we found volume increases in almost every region across the brain with effect sizes (Cohen’s d) ranging from −0.02 to 1.93, corresponding 0% (Left Cerebellum) to 6.7% (Right Amygdala) volume increases. 75% of the 85 regions had a volume increase of at least 0.5 effect size or higher (*t* > 9.8, *p* < 10^−12^, df = 385, Supplementary Table 2A). 246 patients received RUL ECT placement only, 79 bitemporal (BT) only, and 61 individuals first started with RUL and were later switched to BT (MIX) treatment. The overall volume changes were higher in the BT and MIX groups than in the RUL group (mean volume increase: 3.5% ± 1.7%, 3.4% ± 2.1% vs 1.7% ± 1.9%). The BT and MIX had larger number of ECT sessions on average (RUL: 10.9 ± 4.4; BT: 14.3 ± 6.2; MIX: 16.4 ± 5.3) [17]. In addition, independent of the number of ECT sessions, BT and MIX also had higher average EF amplitude in the brain (RUL: 49.0 ± 8.7 V/m; BT: 91.8 ± 15.1 V/m; MIX: 68.7 ± 13.4 V/m). Our results replicated our previous findings in patients with RUL placement [18], extended to other types of electrode placements, and demonstrated a strong correlation across the regions between average EF and volume change in all three groups separately and combined (Fig. 1, Spearman correlations, RUL: *r* = 0.39, *p* = 0.0002; BT: *r* = 0.56, *p* = 1.8 × 10^−8^, MIX: *r* = 0.47, *p* = 5.2 × 10^−6^, df = 83). Several regions showed strong correlations between EF and volume change across individuals even if corrected for age and number of ECT sessions. In agreement with our previous study [18] left hippocampus and amygdala showed the strongest relationship (false discovery rate (FDR) corrected: L Hippocampus: *t*_EF_ = 7.03, *p*_FDR_ = 4 × 10^−10^, Left Amygdala: *t*_EF_ = 9.18, *p*_FDR_ = 2 × 10^−^^16^, Supplementary Table 3A). This relationship remained the same if we removed the 151 individuals with RUL who were the participants of the previous publication [18], (Supplementary Table 3B).

### Unsupervised multivariate analysis

We performed six separate independent PCAs - three for volume changes and three for EF distribution, each corresponding to the different placements. The outcomes were as follows:

#### Volume changes

**Fig. 2:**
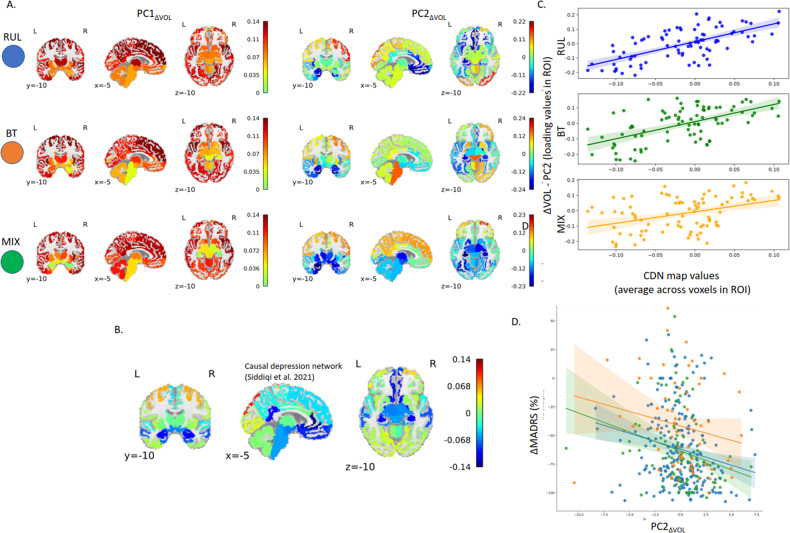
Unsupervised multivariate analysis was conducted using Principal Component Analysis (PCA) on the ΔVOL values. **A** The outcomes (loadings of PCA) are displayed in three rows: the top row represents the findings for subjects with RUL placement, the middle row shows results for patients with BT placement, and the bottom row illustrates subjects with MIX placement. The first component (PC1_ΔVOL_; left side) shows the main effect. The spatial distribution of the second PC of the ΔVOL (PC2_ΔVOL_) was very similar to the Causal Depression Network (CDN) recently reported by Siddiqi et al. (2021) regardless of the electrode placement (**B**) The CDN’s spatial distribution is depicted for visual comparison at the same coronal, sagittal and axial sections, respectively. **C** The scatterplot to illustrate the close similarity between CDN and PC2_ΔVOL_across all types of electrode placements. **D** The more similar the pattern was to the Causal Depression Network the better the clinical outcome was.

In agreement with previous findings [17] the first PC (PC1_ΔVOL_) (Fig. 2A left) was responsible for 42%, 42%, and 41% of the variance in the volume changes in the RUL, BT, and MIX groups, respectively. This 42% variance indicated a strong intra-individual cross-correlation in regional volume increase. The loadings of this main effect showed spatial similarity with the CDN (RUL: *r* = 0.44, BT: *r* = 0.50, MIX: *r* = 0.46) even though it was an unsupervised finding. The second PC (PC2 _ΔVOL_) (Fig. 2A right) was responsible for 6%, 8%, and 11% of the variance and the loading was spatially very similar to the CDN (RUL: *r* = 0.65, BT: *r* = 0.58, MIX: *r* = 0.40, Fig. 2C). Much like the CDN, the PC2_ΔVOL_ networks exhibited lower loadings (relative volume changes) in regions such as the ventromedial prefrontal gyrus, rostral anterior cingulate cortex (rACC), subgenual cingulate, posterior cingulate cortex, hippocampus, amygdala, and entorhinal cortex. Conversely, heightened numbers were observed in the inferior and superior parietal cortex, lateral occipital areas, and the right caudal middle frontal gyrus (a.k.a. frontal eye field). The permutation tests (Supplementary material) indicate that these correlation values are much higher than would be expected from random volume increases generated by shuffling the baseline images across subjects (*p* < 0.0001). Moreover, testing head-to-head the first and second component with multiple regression “CDN values” ~PC1 + PC2, indicated a consistently stronger relationship across the permutations, meaning that when already accounted for the effect of the PC2, there is no evidence that either the PC1 substantially aids modeling the CDN (see supplementary materials, Supplementary Fig. 5).

#### Volume change PC2 _ΔVOL_ and not PC1 _ΔVOL_ correlates with clinical response

Critical to our investigation, our multiple regression analysis ΔMADRS ~ PC1_ΔVOL_ + PC2_ΔVOL_ + age + nECT indicated that PC2 _ΔVOL_, with its remarkable similarity to the CDN, had a significant correlation with clinical response (β _PC1_ = −0.0015, *t*_PC1_ = −0.51, *p* = 0.61; β _PC2_ = −0.016, *t*_PC2_ = −2.35, *p* = 0.019, Fig. 2D, Supplementary Table 4). The more similar the volume change was with the PC2 _ΔVOL_ the better the clinical outcome. Please note that in this and subsequent analyses, when referring to PC1_ΔVOL_, PC2_ΔVOL_, PC1_EF_, and PC2_EF_, we are specifically addressing the expression of the Principal Components (referred to as PC scores) rather than their loadings (as it was in the previous sections). These PC scores are obtained by linearly combining the loadings with their corresponding regional values.

#### EF amplitude

**Fig. 3:**
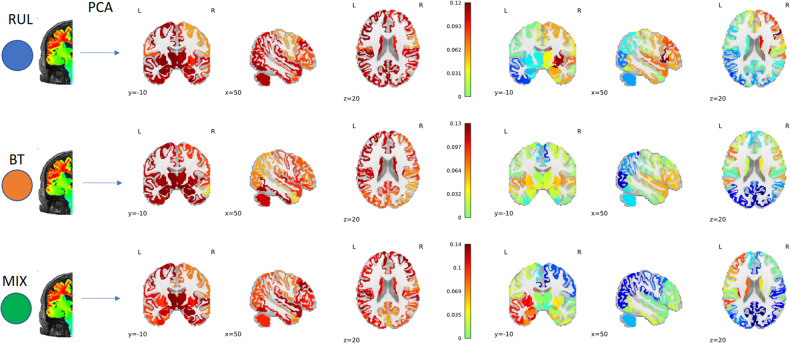
Unsupervised multivariate analysis was conducted using Principal Component Analysis (PCA) on the EF values. The outcomes (loadings of PCA) are displayed in three rows: the top row represents the findings for subjects with RUL placement, the middle row shows results for patients with BT placement, and the bottom row illustrates subjects with MIX placement. The first component (PC1_EF_; left side) shows the main effect. The second PC of the EF (PC2_EF_) reflects the spatial distribution stemming from the different electrode placements.

The first PC1_EF_ (Fig. 3 left) was responsible for 70%, 65%, and 57% of the variance in the EF amplitude in the RUL, BT and MIX groups, respectively. This high variance in the first PC1_EF_ indicated a strong intra-individual correlation across the brain regions, meaning that individuals with high EF had higher EFs across different regions. Therefore, this first PC1_EF_ represented an overall EF magnitude across subjects, which was due to individual differences in brain and head anatomy, including the amount of cerebrospinal fluid and fat tissue. The second PC2_EF_ (Fig. 3 right) was responsible for 7%, 10%, and 24% of the variance, respectively. The spatial distribution of the second PC2_EF_ reflected the electrode placement, showing higher loading near the electrode locations. The loadings of PC2_EF_ did not show any significant correlation with the CDN in RUL and BT. In the MIX group, the PCA analysis indicated that the main (PC1_EF_) and electrode effect (PC2_EF_) was more interleaved, reflecting in the lower and higher variances in the first and second PC_EF_. This was also reflected in its loading structure. Overall, none of the PC_EF_s from the EF amplitudes showed any correlation with the CDN once it was corrected for the spatial coordinates (Supplementary Tables 5A–C, Supplementary Fig. 6).

#### EF amplitude PC1_EF_ and not PC2_EF_ correlates with clinical response

A multiple regression analysis ΔMADRS ~ PC1_EF_ + PC2_EF_ + age + nECT indicated that PC1_EF_, representing overall EF strength, had a significant correlation with clinical response (β_PC1_ = 0.005, *t*_PC1_ = 2.11, *p* = 0.036; β_PC2_ = −0.001, *t*_PC2_ = −0.19, *p* = 0.85, Supplementary Table 6A). The higher the EF amplitude in general was associated with inferior clinical response. PC1_EF_ and PC2_ΔVOL_ negatively correlates (*r* = −0.29, *p* = 6 × 10^−^^9^, df = 384) across the individuals, implying that higher overall EF amplitude in the human brain was associated with lower expression of the PC2_ΔVOL_, which we established to be associated with good clinical effect. Multivariate analysis of ΔMADRS ~ PC1_EF_ + PC2_ΔVOL_ + age + nECT showed that while the effect of PC1_EF_ was not significant, the PC2_ΔVOL_ remained significant (β_PC1EF_ = 0.004, *t*_PC1EF_ = 1.64, *p* = 0.1; β_PC2ΔVOL_ = −0.014, *t*_PC2ΔVOL_ = −1.96, *p* = 0.05; Supplementary Table 6B), indicating tighter association of the clinical outcome with ΔVOL. We complemented our analyses above by conducting linear mixed models by adding sex and electrode placement as fixed effects and site as a random effect, but it did not change the results (Supplementary Material).

### The number of ECT sessions

The extensive changes observed across the brain and the substantial explained variance (~40%) suggest that the PC1_ΔVOL_ might reflect the main effect of ECT. We tested if the number of ECT sessions correlated with PC1_ΔVOL_ and we found a significant positive correlation (*r* = 0.16, *p* = 0.001, df = 384) indicating a dose response relationship. Likewise, the inverse correlation between PC2_ΔVOL_ and the number of ECT sessions was anticipated (*r* = −0.24, *p* < 0.0001, df = 384), as a higher number of sessions was associated with poorer outcomes due to the observational nature of the study (as detailed earlier in the methodological considerations).

### Laterality

Finally, we repeated the same multivariate analyses on the right and the left side of the brain independently (42 ROIs in each hemisphere). We found this necessary for two reasons. First, there is a large literature indicating differences among hemispheres in mood regulation [33]. Second, both the map reported by Siddiqi et al. and the bilateral placement maps are highly symmetrical which could lead to a spurious overestimation of the results.

The loadings of the PCs acquired on the entire brain appeared highly symmetrical (Supplementary Fig. 7). As expected, the PC_EF_s of the EF in the RUL setting were the least symmetrical, but the volume changes were more symmetrical across different electrode placements. When we ran the PCAs separately for the right and left hemispheres (PCA_right_, PCA_left_), we found that the results were very similar for the PCA_right_ (Supplementary Figs. 8 and 6, *r*: 0.72–0.99). We found that the second PC2_ΔVOL_ of volume change highly correlated with the CDN and with the clinical response (Supplementary Material). The second PC2_ΔVOL_ of volume change also correlated highly with the loadings of the original PCA (Supplementary Fig. 9). The PCA_left_ led to a similar loading structure in their first PCs of the EF and volume changes (main effect, *r* = 0.61–0.95), but the second PCs were different in RUL and BT (*r* = 0.01, 0.47, respectively) and volume change did not correlate with clinical outcome (Supplementary material).

## Discussion

Our study is a comprehensive multivariate analysis of 386 patients with depression who underwent ECT and longitudinal neuroimaging. Our multivariate non-supervised analysis (PCA) revealed a hidden pattern in volume change that was correlated with clinical outcome. The same pattern was found independently in the three separate groups, RUL, BT and MIX electrode placements, and this pattern showed striking similarities to the common causal circuit recently published in a study of large cohort of independent samples of depressed patients [3, 4]. This network consists of cortical and sub-cortical areas previously implicated in depression or emotion regulation, such as the subgenual cingulate cortex, dorsolateral prefrontal cortex, ventromedial prefrontal cortex and hippocampus.

Initial studies of ECT effect on structural neuroimaging on limited sample sizes (*N* ~ 20) often focused on hippocampus increase. As it became clear later, more widespread volume changes with moderate effect sizes were present in these samples, but due to the limited sample size, it did not reach statistical significance [15]. After establishing the GEMRIC consortium [15] and collecting hundreds of individuals, ECT studies repeatedly and consistently showed increased volume in both cortical and subcortical regions [16–18]. The GEMRIC data also demonstrated that the volume increase correlated with the EF amplitude in RUL electrode placement [18]. This relationship between EF and volume change was recently replicated in an independent cohort [19]. The current findings further confirm this relationship in an array of groups with different and often mixed electrode placements (RUL, BT, and MIX). The previous findings were replicated, and the weighted mixing of the EF values according to the number of ECT sessions on different electrode placements proved to be a useful way to calculate the effect of EF on volume change (MIX electrode placement). Our current study further confirms that EF modeling, despite its limitation [34], is a useful technique to estimate EF.

### Multivariate analysis and clinical effect

We found a spatial pattern in the volume changes on top of the main effect which showed distinct similarities to the CDN map reported by Siddiqi et al and was responsible for approximately 6%–11% of the total variance. Most importantly, the more this pattern was expressed, the better the clinical outcome was. Our approach had two vital aspects that could further boost confidence about the validity of these findings. First, it was unsupervised and data-driven, to avoid overfit and no information about the CDN was used to conduct our analysis. Second, we analyzed the three electrode placement groups separately as independent samples. We not only received equivalent results across distinct groups, but these results were highly similar to the common causal circuit reported by Siddiqi et al. As reported in patients treated with DBS and TMS, individual similarity to this map correlated with antidepressant outcome in ECT.

A set of interconnected areas, with sometime opposite signs in their relationship, is reliably implicated in association with depression. In addition to the original discovery, a very recent lesion mapping study in patients with multiple sclerosis resulted in a very similar map, showing close correlation with depression rates. While our methodology deviates from traditional lesion mapping, as it is measuring structural volume change patterns, the second principal component of the variance shows a distinct similarity with the networks reported previously. The brain wide volume changes which failed to associate with clinical response likely includes both epiphenomena and antidepressant volumetric changes [17] Our results suggest that the antidepressant volumetric changes are relatively hidden (PC2_ΔVOL_) behind the main volumetric effect of ECT (PC1 _ΔVOL_). This explains why previous univariate approaches failed to detect correlations between volume change and clinical response. Without decomposing the volume changes to a main effect (PC1 _ΔVOL_), which is responsible for most of the variance, and to an orthogonal pattern (PC2 _ΔVOL_), this second pattern would remain hidden. Our results indicate that only this second pattern of change, which is similar to the pattern obtained by Siddiqi et al, has clinical relevance. The second component explains a relatively small amount of variance (6–11%) compared to the first component (~40%). This raises the question of what, if anything, PC1 _ΔVOL_ is associated with. Earlier findings also suggest that certain volume increases can lead to cognitive side effects often linked with ECT [35]. Unfortunately, we were unable to examine whether PC1_ΔVOL_ and PC2_ΔVOL_ can differentiate these effects due to the unavailability of harmonized neurocognitive data across different sites. The overall interpretation of these two volumetric PCs was reinforced by the observed correlations between their values and the number of ECT sessions. The positive relationship between PC1_ΔVOL_ and the number of ECT sessions suggests a dose response relationship that is independent of the clinical response. Although this correlation is modest, it is important to acknowledge that the number of ECT sessions serves as a rudimentary approximation of the overall ECT dosage, which can vary individually based on the seizure threshold. On the contrary, the inverse correlation between PC2_ΔVOL_ and the number of ECT sessions arises from an indirect association, given that a higher number of ECT sessions was linked with poorer outcomes in this observational cohort of patients (as outlined in the methodological considerations above).

Considering the convergence of findings between TMS and DBS-based CDN, along with our PC2_ΔVOL_, it prompts the question of whether there exists a volumetric effect of DBS and TMS. Although human studies reporting structural changes after DBS in depression are limited [36], animal studies have demonstrated that deep brain stimulation of the ventromedial prefrontal cortex leads to a distant effect, resulting in increased hippocampal and thalamic volumes [37]. In the case of TMS, there is more evidence for macroscopically measurable effects across the brain. It has been demonstrated that as few as five TMS treatments can lead to measurable macroscopic changes in the superior temporal cortex [38]. Subsequent studies [39, 40] implicated the rACC not only in volume changes but also in its correlation with clinical outcomes. The rACC is one of the peak regions in the PC2 and CDN, exhibiting a notable convergence with both this region and the prior literature [41]. It is important to acknowledge a noteworthy contradiction here, as our results show the opposite sign compared to previous TMS studies. However, our results indicate a “residual” effect over the main effect, which is an overall increase in ECT. Therefore, careful interpretation is needed when comparing these results with distinct methodologies.

Furthermore, multivariate analysis of the EF revealed two components: the first represented the main effect, the overall strength of the EF across the brain, and the second was specific to the spatial particularities of the electrode placement. The first component that reflected the overall EF strength correlated with clinical response, indicating that higher EF was associated with worse outcome. We also found that the higher the first component of EF was expressed, the lower the CDN expression in the patient (the second component of the volume change). In a classical sense [42] our multiple regression analysis between clinical effect and the principal components of the EF and ΔVOL might imply that the high EF was mediated through a lower expression of the beneficial pattern, leading to a less than optimal outcome. As there are several limitations to deduct causal inference from classical mediation analysis, we point out here the need of prospective studies with preselected parameters to determine true cause and effect relationships in the future. The observation that high general EF was associated with worse clinical outcome is counterintuitive first, but supported by one previous study in a smaller, and only in BT treated cohort [21], and might indicate that more focal or low amplitude treatments would be more beneficial. This seemingly contradicts some of the clinical observations in recent studies of RUL patients where the amplitude of the current was modified and was found that below certain range the clinical effect was insufficient [22]. However, these tested values (600, 700 and 800 mA) were below the range we use in current clinical practice and constitute the database analyzed in this article (800 and 900 mA). These results suggest that too low or too high EF might be equally suboptimal (inverted U hypothesis of strength of EF and antidepressant outcomes) [43]. The optimal strength of EF and its proper spatial distribution is an intriguing new direction that must be systematically investigated in the future.

Finally, PCA on each hemisphere independently showed that the first PC, representing the main effect, was similar regardless of the analytical approach (whole brain, right or left side, Supplementary Fig. 9). There were, however, hemispheric differences in the second PC_ΔVOL_s: whole-brain PCA results were only replicated on the right side. This implies that neuroplastic changes associated with clinical outcomes were more robust on the right side [44, 45].

The study has some noteworthy limitations. First there are significant methodological differences between the original study and the current approach. While the original approach was a voxel based lesion network mapping, this is ROI based multivariate structural analysis. It’s worth noting the potential drawback to this method is that certain aspects of the topography in the Siddiqi et al. map may be compromised when transformed into larger ROIs covering multiple functional regions. Secondly, our analysis was limited to identifying associations between imaging results and depression outcomes. Therefore, we were unable to fully demonstrate the specificity of our findings or to test other associations. Thirdly, we recognize that it would have been ideal to model not only the E-field but also the ECT dose. Unfortunately, only the number of ECT sessions was available for this cohort, serving as a crude proxy for estimating the overall ECT dose. Lastly, the variability in potential image quality and clinical practices among different sites could have introduced some level of noise into the analysis. It was evident that certain sites predominantly utilized either RUL or BT, thereby introducing a potential bias in electrode placement across the various sites.

In summary, this current report is a significant step forward in understanding the direct electrical stimulation aspect of ECT and its effect on brain volume changes and clinical outcomes. However, our findings, in their present state, do not offer direct clinical guidance, as we have yet to fully characterize the relationship between ECT dosage, electrode placement, network formation timeline, and specificity.

To address this gap, we must investigate the dose-response dynamics of volume change pattern formation, considering factors such as (1) EF strength, (2) electrode placement, (3) dosage, and (4) seizure patterns, all of which influence volume changes. This understanding is crucial for designing prospective clinical studies that aim for optimal benefit. The studies require ECT machines capable of varying current amplitude, random electrode placement assignment to mitigate site-specific effects, and ideally a consistent number of stimuli. Our deepening grasp of this domain can guide the development of well-informed studies and methodologies to refine treatments.

To establish causality, we can test if parameter settings that enhance CDN formation in volume changes lead to improved treatment effects. Concurrently, future ECT studies should explore similar networks in psychotic disorders to discern whether a transdiagnostic signature exists or if patterns differ across disorders.

Overall, the revelation that the same neural network associated with clinical benefits in TMS and DBS is also implicated in ECT offers promise. It suggests that delving further into ECT-related clinical networks could shed light on these other treatment modalities as well.

### Supplementary information

Supplementary Material

## Data Availability

Raw data cannot be made available publicly because we do not have consent or ethical approval for the public release and the data cannot be anonymized. The data are stored on a secure centralized server at the University of Bergen, Norway. Participating GEMRIC sites have access to the raw data according to specific data policy and safety rules of the consortium, and in accord with the approval from the ethical committee. The GEMRIC consortium welcomes new members who are interested in the neuroimaging research of ECT. We hold board meetings twice a year when new members can apply to join and gain access to the database available on the GEMRIC servers. For more about the application process please visit https://mmiv.no/how-to-join-gemric/ or write to Leif Oltedal (leif.oltedal@uib.no). General information about the consortium can be found on the following website: https://mmiv.no/gemric/. For transparency and reproducibility, the entire analytical approach is uploaded to https://github.com/argyelan/Publications/tree/master/VOLUME-CHANGE-PCA.

## References

[1] UK ECT Review Group. (2003). Efficacy and safety of electroconvulsive therapy in depressive disorders: a systematic review and meta-analysis. Lancet.

[2] Mutz J, Vipulananthan V, Carter B, Hurlemann R, Fu CHY, Young AH (2019). Comparative efficacy and acceptability of non-surgical brain stimulation for the acute treatment of major depressive episodes in adults: systematic review and network meta-analysis. BMJ.

[3] Siddiqi SH, Schaper FLWVJ, Horn A, Hsu J, Padmanabhan JL, Brodtmann A (2021). Brain stimulation and brain lesions converge on common causal circuits in neuropsychiatric disease. Nat Hum Behav.

[4] Siddiqi SH, Kletenik I, Anderson MC, Cavallari M, Chitnis T, Glanz BI (2023). Lesion network localization of depression in multiple sclerosis. Nat Ment Health.

[5] Morawetz C, Riedel MC, Salo T, Berboth S, Eickhoff SB, Laird AR (2020). Multiple large-scale neural networks underlying emotion regulation. Neurosci Biobehav Rev.

[6] Huang Y, Liu AA, Lafon B, Friedman D, Dayan M, Wang X et al. Measurements and models of electric fields in the in vivo human brain during transcranial electric stimulation. eLife (2017); **6**. 10.7554/eLife.18834. 10.7554/eLife.18834 PMC5370189 28169833

[7] Bai S, Loo C, Al Abed A, Dokos S (2012). A computational model of direct brain excitation induced by electroconvulsive therapy: comparison among three conventional electrode placements. Brain Stimul.

[8] Bai S, Gálvez V, Dokos S, Martin D, Bikson M, Loo C (2017). Computational models of bitemporal, bifrontal and right unilateral ECT predict differential stimulation of brain regions associated with efficacy and cognitive side effects. Eur Psychiatry.

[9] Deng Z-D, Lisanby SH, Peterchev AV (2015). Effect of anatomical variability on electric field characteristics of electroconvulsive therapy and magnetic seizure therapy: a parametric modeling study. IEEE Trans Neural Syst Rehabil Eng.

[10] Bliss TV, Lomo T (1973). Long-lasting potentiation of synaptic transmission in the dentate area of the anaesthetized rabbit following stimulation of the perforant path. J Physiol.

[11] Hesse GW, Teyler TJ (1976). Reversible loss of hippocampal long term potentiation following electronconvulsive seizures. Nature.

[12] Huang Y-Z, Edwards MJ, Rounis E, Bhatia KP, Rothwell JC (2005). Theta burst stimulation of the human motor cortex. Neuron.

[13] Ito M, Seki T, Liu J, Nakamura K, Namba T, Matsubara Y (2010). Effects of repeated electroconvulsive seizure on cell proliferation in the rat hippocampus. Synapse.

[14] Zhao C, Warner-Schmidt J, Duman RS, Gage FH (2012). Electroconvulsive seizure promotes spine maturation in newborn dentate granule cells in adult rat. Dev Neurobiol.

[15] Oltedal L, Bartsch H, Sørhaug OJE, Kessler U, Abbott C, Dols A (2017). The Global ECT-MRI Research Collaboration (GEMRIC): establishing a multi-site investigation of the neural mechanisms underlying response to electroconvulsive therapy. Neuroimage Clin.

[16] Oltedal L, Narr KL, Abbott C, Anand A, Argyelan M, Bartsch H (2018). Volume of the human hippocampus and clinical response following electroconvulsive therapy. Biol Psychiatry.

[17] Ousdal OT, Argyelan M, Narr KL, Abbott C, Wade B, Vandenbulcke M (2020). Brain changes induced by electroconvulsive therapy are broadly distributed. Biol Psychiatry.

[18] Argyelan M, Oltedal L, Deng Z-D, Wade B, Bikson M, Joanlanne A et al. Electric field causes volumetric changes in the human brain. eLife 2019;8. 10.7554/eLife.49115. 10.7554/eLife.49115 PMC6874416 31644424

[19] Deng Z-D, Argyelan M, Miller J, Quinn DK, Lloyd M, Jones TR (2022). Electroconvulsive therapy, electric field, neuroplasticity, and clinical outcomes. Mol Psychiatry.

[20] Takamiya A, Bouckaert F, Laroy M, Blommaert J, Radwan A, Khatoun A (2021). Biophysical mechanisms of electroconvulsive therapy-induced volume expansion in the medial temporal lobe: a longitudinal in vivo human imaging study. Brain Stimul.

[21] Fridgeirsson EA, Deng Z-D, Denys D, van Waarde JA, van Wingen GA (2021). Electric field strength induced by electroconvulsive therapy is associated with clinical outcome. Neuroimage Clin.

[22] Abbott CC, Quinn D, Miller J, Ye E, Iqbal S, Lloyd M (2021). Electroconvulsive therapy pulse amplitude and clinical outcomes. Am J Geriatr Psychiatry.

[23] Gryglewski G, Lanzenberger R, Silberbauer LR, Pacher D, Kasper S, Rupprecht R (2021). Meta-analysis of brain structural changes after electroconvulsive therapy in depression. Brain Stimul.

[24] Mulders PCR, Llera A, Beckmann CF, Vandenbulcke M, Stek M, Sienaert P (2020). Structural changes induced by electroconvulsive therapy are associated with clinical outcome. Brain Stimul.

[25] Merkel D. Docker: lightweight linux containers for consistent development and deployment. Linux J 2014;239:2. https://www.linuxjournal.com/content/docker-lightweight-linux-containers-consistent-development-and-deployment.

[26] Jovicich J, Czanner S, Greve D, Haley E, van der Kouwe A, Gollub R (2006). Reliability in multi-site structural MRI studies: effects of gradient non-linearity correction on phantom and human data. Neuroimage.

[27] Fischl B, Salat DH, Busa E, Albert M, Dieterich M, Haselgrove C (2002). Whole brain segmentation: automated labeling of neuroanatomical structures in the human brain. Neuron.

[28] Desikan RS, Ségonne F, Fischl B, Quinn BT, Dickerson BC, Blacker D (2006). An automated labeling system for subdividing the human cerebral cortex on MRI scans into gyral based regions of interest. Neuroimage.

[29] Reuter M, Schmansky NJ, Rosas HD, Fischl B (2012). Within-subject template estimation for unbiased longitudinal image analysis. Neuroimage.

[30] O’Connor MK, Knapp R, Husain M, Rummans TA, Petrides G, Smith G (2001). The influence of age on the response of major depression to electroconvulsive therapy: a C.O.R.E. Report. Am J Geriatr Psychiatry.

[31] van Diermen L, van den Ameele S, Kamperman AM, Sabbe BCG, Vermeulen T, Schrijvers D (2018). Prediction of electroconvulsive therapy response and remission in major depression: meta-analysis. Br J Psychiatry.

[32] Socci C, Medda P, Toni C, Lattanzi L, Tripodi B, Vannucchi G (2018). Electroconvulsive therapy and age: age-related clinical features and effectiveness in treatment resistant major depressive episode. J Affect Disord.

[33] Gibson BC, Vakhtin A, Clark VP, Abbott CC, Quinn DK. Revisiting hemispheric asymmetry in mood regulation: implications for rTMS for major depressive disorder. Brain Sci. 2022;12. 10.3390/brainsci12010112. 10.3390/brainsci12010112 PMC8774216 35053856

[34] Sartorius A (2022). Electric field distribution models in ECT research. Mol Psychiatry.

[35] Argyelan M, Lencz T, Kang S, Ali S, Masi PJ, Moyett E (2021). ECT-induced cognitive side effects are associated with hippocampal enlargement. Transl Psychiatry.

[36] Elias GJB, Germann J, Loh A, Boutet A, Pancholi A, Beyn ME, et al. Habenular involvement in response to subcallosal cingulate deep brain stimulation for depression. Front Psychiatry. 2022;13:810777. 10.3389/fpsyt.2022.810777 PMC8854862 35185654

[37] Chakravarty MM, Hamani C, Martinez-Canabal A, Ellegood J, Laliberté C, Nobrega JN, et al. Deep brain stimulation of the ventromedial prefrontal cortex causes reorganization of neuronal processes and vasculature. Neuroimage. 2016;125:422–7. 10.1016/j.neuroimage.2015.10.049 26525655

[38] May A, Hajak G, Gänssbauer S, Steffens T, Langguth B, Kleinjung T, et al. Structural brain alterations following 5 days of intervention: dynamic aspects of neuroplasticity. Cereb Cortex. 2007;17:205–10. 10.1093/cercor/bhj138 16481564

[39] Lan MJ, Chhetry BT, Liston C, Mann JJ, Dubin M. Transcranial Magnetic Stimulation of Left Dorsolateral Prefrontal Cortex Induces Brain Morphological Changes in Regions Associated with a Treatment Resistant Major Depressive Episode: An Exploratory Analysis. Brain Stimulat. 2016;9:577–83. 10.1016/j.brs.2016.02.011 PMC5554068 27017072

[40] Boes AD, Uitermarkt BD, Albazron FM, Lan MJ, Liston C, Pascual-Leone A, et al. Rostral anterior cingulate cortex is a structural correlate of repetitive TMS treatment response in depression. Brain Stimulat. 2018;11:575–81. 10.1016/j.brs.2018.01.029 PMC6136654 29454551

[41] Mayberg HS. Limbic-cortical dysregulation: a proposed model of depression. J Neuropsychiatry Clin Neurosci. 1997;9:471–81. 10.1176/jnp.9.3.471 9276848

[42] Baron RM, Kenny DA (1986). The moderator–mediator variable distinction in social psychological research: conceptual, strategic, and statistical considerations. J Pers Soc Psychol.

[43] Ousdal OT, Brancati GE, Kessler U, Erchinger V, Dale AM, Abbott C (2022). The neurobiological effects of electroconvulsive therapy studied through magnetic resonance: what have we learned, and where do we go?. Biol Psychiatry.

[44] Gainotti G, Caltagirone C, Zoccolotti P (1993). Left/right and cortical/subcortical dichotomies in the neuropsychological study of human emotions. Cogn Emot.

[45] Gainotti G. Emotions and the right hemisphere: can new data clarify old models? Neuroscientist. 2019;25:258–70. 10.1177/1073858418785342 29985120

