# Correction: Electroconvulsive therapy-induced volumetric brain changes converge on a common causal circuit in depression

**DOI:** 10.1038/s41380-023-02358-8

**Published:** 2023-12-05

**Authors:** Miklos Argyelan, Zhi-De Deng, Olga Therese Ousdal, Leif Oltedal, Brian Angulo, Mate Baradits, Andrew J. Spitzberg, Ute Kessler, Alexander Sartorius, Annemiek Dols, Katherine L. Narr, Randall Espinoza, Jeroen A. van Waarde, Indira Tendolkar, Philip van Eijndhoven, Guido A. van Wingen, Akihiro Takamiya, Taishiro Kishimoto, Martin B. Jorgensen, Anders Jorgensen, Olaf B. Paulson, Antoine Yrondi, Patrice Péran, Carles Soriano-Mas, Narcis Cardoner, Marta Cano, Linda van Diermen, Didier Schrijvers, Jean-Baptiste Belge, Louise Emsell, Filip Bouckaert, Mathieu Vandenbulcke, Maximilian Kiebs, René Hurlemann, Peter CR. Mulders, Ronny Redlich, Udo Dannlowski, Erhan Kavakbasi, Michael D. Kritzer, Kristen K. Ellard, Joan A. Camprodon, Georgios Petrides, Anil K. Malhotra, Christopher C. Abbott

**Affiliations:** 1 Feinstein Institutes for Medical Research, Institute of Behavioral Science, Manhasset, NY USA; 2 The Zucker Hillside Hospital, Glen Oaks, NY USA; 3 Noninvasive Neuromodulation Unit, Experimental Therapeutics and Pathophysiology Branch, National Institute of Mental Health, National Institutes of Health, Bethesda, MD USA; 4 Department of Psychiatry and Behavioral Sciences, Duke University School of Medicine, Durham, NC USA; 5 Department of Biomedicine, Faculty of Medicine, University of Bergen, Bergen, Norway; 6 Department of Radiology, Haukeland University Hospital, Bergen, Norway; 7 Department of Clinical Medicine, University of Bergen, Bergen, Norway; 8 Mohn Medical Imaging and Visualization Centre, Department of Radiology, Haukeland University Hospital, Bergen, Norway; 9 Department of Psychiatry and Psychotherapy, Semmelweis University, Budapest, Hungary; 10 Department of Psychiatry, Haukeland University Hospital, University of Bergen, Bergen, Hungary; 11 Department of Psychiatry and Psychotherapy, Central Institute of Mental Health (CIMH), Medical Faculty Mannheim, University of Heidelberg, Heidelberg, Germany; 12 Department of Psychiatry, UMC Utrecht Brain Center, University Medical Center Utrecht, Utrecht, The Netherlands; 13 Amsterdam UMC location Vrije Universiteit Amsterdam, Psychiatry, Neuroscience, Amsterdam, The Netherlands; 14 Department of Neurology, University of California Los Angeles, Los Angeles, CA USA; 15 Department of Psychiatry and Biobehavioral Sciences, University of California Los Angeles, Los Angeles, CA USA; 16 Rijnstate, Arnhem, the Netherlands; 17 Donders Institute for Brain, Cognition and Behavior, Department of Psychiatry, Nijmegen, the Netherlands; 18 Amsterdam UMC location University of Amsterdam, Department of Psychiatry, Amsterdam, The Netherlands; 19 Amsterdam Neuroscience, Amsterdam, The Netherlands; 20 Department of Neuropsychiatry Keio University School of Medicine, Tokyo, Japan; 21 Neuropsychiatry, Department of Neurosciences, Leuven Brain Institute, KU Leuven, Belgium; 22 Hills Joint Research Laboratory for Future Preventive Medicine and Wellness, Keio University School of Medicine, Tokyo, Japan; 23 Psychiatric Center Copenhagen and Department of Clinical Medicine, University of Copenhagen, Copenhagen, Denmark; 24 Neurobiological Research Unit Rigshospitalet and Department of Clinical Medicine, University of Copenhagen, Copenhagen, Denmark; 25 Service de Psychiatrie et Psychologie Médicale, Centre Expert Dépression Résistante, Fondation Fondamental, CHU Toulouse, ToNIC, Toulouse NeuroImaging Center, Univerité de Toulouse, Inserm, UPS, Toulouse, France; 26 ToNIC, Toulouse NeuroImaging Center, Univeristé de Toulouse, Inserm, UPS, Toulouse, France; 27 Department of Social Psychology and Quantitative Psychology, Universitat de Barcelona-UB, Barcelona, Spain; 28 Bellvitge Biomedical Research Institute-IDIBELL, Department of Psychiatry, Bellvitge University Hospital, Barcelona, Spain; 29 CIBERSAM, Carlos III Health Institute, Madrid, Spain; 30 Sant Pau Mental Health Research Group, Institut d’Investigació Biomèdica Sant Pau (IIB-Sant Pau), Hospital de la Santa Creu i Sant Pau, Barcelona, Spain; 31 Department of Psychiatry and Forensic Medicine, School of Medicine Bellaterra, Universitat Autònoma de Barcelona, Barcelona, Spain; 32 Department of Psychiatry, Collaborative Antwerp Psychiatric Research Institute (CAPRI), Faculty of Medicine and Health Sciences, University of Antwerp, Antwerp, Belgium; 33 Psychiatric Center Bethanie, Andreas Vesaliuslaan 39, 2980 Zoersel, Belgium; 34 University Psychiatric Center Duffel, Stationstraat 22, Duffel, 2570 Belgium; 35 Department of Psychiatry, Radboud University Medical Centre, P.O. Box 9101, 6500 HB Nijmegen, The Netherlands; 36 Geriatric Psychiatry, University Psychiatric Center-KU Leuven, Leuven, Belgium; 37 School of Medicine & Health Sciences University Hospital Oldenburg, Oldenburg, Germany; 38 Department of Psychiatry and Psychotherapy University Hospital Bonn, Bonn, Germany; 39 Department of Psychology, University of Halle, Halle, Germany; 40 German Center for Mental Health (DZPG), Site Jena-Magdeburg-Halle, Halle, Germany; 41 Department of Translational Psychiatry, University of Muenster, Muenster, Germany; 42 Department of Mental Health, University of Muenster, Muenster, Germany; 43 Department of Psychiatry, Massachusetts General Hospital, Harvard Medical School, Boston, MA USA; 44 Department of Psychiatry, University of New Mexico, Albuquerque, NM USA

**Keywords:** Predictive markers, Depression

Correction to: *Molecular Psychiatry* 10.1038/s41380-023-02318-2, published online 20 November 2023

In the original version of this article, the given and family names of Erhan Kavakbasi were incorrectly reversed. The name was displayed correctly in all versions at the time of publication. The original article has been corrected.

