# Supplementary material for: Electroconvulsive therapy-induced volumetric brain changes converge on a common causal circuit in depression
Source: Mol Psychiatry. 2023 Nov 20;29(2):229–37. doi: 10.1038/s41380-023-02318-2 (PMC11116108; doi:10.1038/s41380-023-02318-2)
Supplement: Supplementary file 1 — Supplementary Material [file 41380_2023_2318_MOESM1_ESM.docx]

Supplementary material

Spatial similarity calculations

The spatial distribution of the principal components' loadings can be compared with that of the Causal Depression Network (CDN). While a straightforward approach might involve correlating these values, it's important to note that traditional parametric correlation tests are not applicable in this case, as the regional values are not independent. To address this, we conducted two distinct permutation tests.

First, we independently permuted the regional values across subjects. This involved randomly assigning the value ΔVOL_ij_ from the ith region and jth subject to ΔVOL_ik,_ another kth subject in the same region, for all ROIs. Following this permutation, we conducted PCA analysis and determined if the original spatial similarity metric exceeded the 99.86 percentile (adjusted for multiple comparisons using Bonferroni correction for 36 tests with p<0.05, resulting in a threshold of 0.000136) of the permuted values.

The second permutation test focused solely on the volume changes. In this analysis, we permuted the baseline images, calculated changes by randomly pairing individuals, and subsequently performed PCA. Both permutation analyses consisted of 1000 iterations each. The calculations are documented at https://github.com/argyelan/Publications/tree/master/VOLUME-CHANGE-PCA in Permutation_PCA.ipynb. The results are shown in Supplementary Figure 5. The findings collectively suggest that the observed correlation greatly exceeds what would be anticipated from random sampling (p<0.001).

Spatial coordinates as covariates

We conducted a multiple regression of the "CDN values" ~ abs(X) + Y + Z, where X, Y, and Z were the coordinates across the LR (left-right), PA (posterior-anterior) and the IS (inferior-superior) axes, respectively. The Siddiqi map values showed strong correlations across the spatial dimensions of the regions, especially across the posterior-anterior and inferior-superior axis (F_3,81_ = 19.6, p = 1x10^-9^; t_Xabs_ = 5.1, p < 0.0001, t_Y_ = -2.2, p = 0.03, t_Z_ = 5.2, p < 0.0001), indicating higher values on the lateral and on the superior areas. The solid spatial similarities between the CDN and the main (loadings of PC1_ΔVOL_) and the secondary effect (loadings of PC2 _ΔVOL_) raised the question if these maps were only reflecting gross similarities across the posterior-anterior or inferior-superior direction. One could argue that both RUL and BT had a higher impact on the superior and lateral areas, resulting in a more reliable volume change in these regions leading to a PC that had grossly matching spatial distribution with the CDN. We tested this hypothesis by conducting a multiple regression with spatial coordinates of the regions as confounders: "CDN values" ~ PC1 + PC2 + X + Y + Z in all three groups (*RUL*: F_5,79_ = 19.07, p = 2x10^-12^; t_PC1_ = 2.13, p = 0.04; **t_PC2_ = 4.66, p = 1x10^-5^**; t_Xabs_ = 2.25, p = 0.03; t_Y_ = 1.10, p = 0.27; t_Z_ = -0.38, p = 0.70, *BT*: F_5,79_ = 19.18, p = 2x10^-12^; t_PC1_ = 2.07, p = 0.04; **t_PC2_ = 4.37, p = 4x10^-5^**; t_Xabs_ = 3.44, p = 0.0009; t_Y_ = -1.78, p = 0.08; t_Z_ = 0.84, p = 0.40, and *MIX*: F_5,79_ = 12.8, p = 4x10^-9^; t_PC1_ = 1.15, p = 0.26; t_PC2_ = 0.69, p = 0.49; t_Xabs_ = 4.25, p = 6x10^-5^; t_Y_ = -2.43, p = 0.02; t_Z_ = 2.32, p = 0.023). In both RUL and BT groups, the results equivocally identified that PC2 showed highly significant similarities that could not be explained by gross anatomical similarities (Supplementary Table 4 A, B, and C). We acknowledge that the above calculations may overestimate the effect due to the potential violation of independence between regions. Therefore, we employed a similar approach using permuted Principal Components (PCs) and computed the distributions of the t statistics. Initially, we held PC2_ΔVOL_ constant (not permuted) and permuted PC1_ΔVOL_ to obtain a distribution for t of PC1_ΔVOL_. Subsequently, we held PC1_ΔVOL_ constant and permuted PC2_ΔVOL_. The permutation results further supported that the PC_ΔVOL_ exhibit a higher degree of similarity to the CDN than what would be anticipated solely based on basic spatial dimensions (for example that the lateral and upper regions are simply more involved) in the RUL and BT cases. However, akin to the parametric tests, this similarity diminished in the MIX case and became non-significant. The results are illustrated in Supplementary Figure 6 upper panel. Similar permutation of PC1_EF_ and PC2_EF_ resulted in showing that there is no significant similarity in any of the loading structures after corrected for the spatial dimensions (Supplementary Figure 6 B).

Detailed analysis of the PCA conducted only on the right side

We follow the analysis steps of PCA_whole_brain_:

Volume changes: We repeated the PCA analysis only on the right side on 42 ROIs. The first PC (Supplementary Figure 8A left) was responsible for 44, 45, and 45% of the variance in the volume changes in the RUL, BT, and MIX groups, respectively. This 45% variance indicated a strong intra-individual cross-correlation in regional volume increase and its value is similar to the PCA_whole_brain_. The loadings of this main effect showed spatial similarity with the causal depression network (CDN) (RUL: r = 0.42, BT: r = 0.60, MIX: r = 0.44, df=40) even though it was an unsupervised finding. The second PC (Supplementary Figure 8A right) was responsible for 8%, 10%, and 11% of the variance and the loading was spatially very similar to the CDN (RUL: r = 0.62; BT: r = 0.69, MIX: r = 0.46; df=40, Supplementary Figure 8C). The effect sizes of similarity of loadings of the second component were higher in all groups than in the first component.

Volume change PC2 and not PC1 correlates with clinical response: Our multivariate regression analysis ΔMADSR ~ PC1_ΔVOL_ + PC2_ΔVOL_ + age + nECT indicated that PC2, with its remarkable similarity to the CDN, had a significant correlation with clinical response (F_4,381_ = 15.91, p = 5x10^-12;^ t_PC1_ = -0.78, p = 0.44; **t_PC2_ = -2.23, p = 0.026**; t_age_ = -5.94, p < 0.0001; t_nECT_ = 3.00, p = 0.003, Figure 2D). The more similar the volume change was with the PC2 the better the clinical outcome.

EF amplitude: The first PC (Supplementary Figure 8B left) was responsible for 67%, 61%, and 62% of the variance in the EF amplitude in the RUL, BT and MIX groups, respectively. The second PC (Supplementary Figure 8B right) was responsible for 8%, 11%, and 17% of the variance, respectively. The spatial distribution of the second PC reflected the electrode placement, showing higher loading near the electrode locations. The loadings of PC2 did not show any significant correlation with the CDN in RUL and BT. In the MIX group the PCA analysis indicated that the main (PC1) and electrode effect (PC2) is more interleaved, reflecting in the lower and higher variances in the first and second PC. This was also reflected in its loading structure. Overall, none of the PCs from the EF amplitudes showed any correlation with the CDN once it was corrected for the spatial coordinates.

EF amplitude PC1 and not PC2 correlates with clinical response: A multivariate regression analysis ΔMADSR ~ PC1_EF_ + PC2_EF_ + age + nECT indicated that PC1, the one that represents the overall EF strength, had a significant correlation with clinical response (F_4,381_ = 15.50, p = 9x10^-12;^ **t_PC1_ = -2.05, p = 0.04**; t_PC2_ = 0.07, p = 0.94; t_age_ = -5.36, p < 0.0001; t_nECT_ = 3.27, p = 0.001). The higher the EF amplitude the worse the clinical response.

Does EF interfere with volume changes to influence clinical effect?

PC1_EF_ and PC2_ΔVOL_ negatively correlates (r = -0.19, p = 0.0002, df = 384) across the individuals, implying that higher overall EF amplitude in the human brain is associated with lower expression of the PC2_ΔVOL_, which we established to be associated with good clinical effect. The question is if high PC1_EF_ negative effect is mediated through the volume changes, or is independent. The multivariate analysis of ΔMADSR ~ PC1_EF_ + PC2_ΔVOL_ + age + nECT shows that the PC1_EF_ effect is mediated through PC2_ΔVOL_ (F_4,381_ = 16.64, p = 1x10^-12;^ t_PC1EF_ = 1.77, p = 0.08; **t_PC2ΔVOL_ = -2.00, p = 0.04**; t_age_ = -5.36, p < 0.0001; t_nECT_ = 2.75, p = 0.006).

Overall, the results from PCA_right_ and PCA_whole_brain_ are very similar. The loadings were almost identical in the second PC: with Pearson correlation r of 0.97, 0.99, and 0.99 for RUL, BT, and MIX, respectively (df=40).

PCA_left_ had very similar first PCs (Supplementary Figure 9); however, the most critical ΔVOL PC2 deviated from PCA_right_ and PCA_whole_brain_, and showed no significant correlation with 1) CDN (RUL: r = 0.22, p = 0.16; BT: r = -0.14, p = 0.38, df = 40) 2) or with clinical response (t=1.36, p=0.17).

Complementary analyses adding sex and electrode placement as fixed effect and site as a random effect (for more details see code provided)

ΔMADRS ~ PC1_ΔVOL_ + PC2_ΔVOL_ + age + nECT + sex + placement + site

| Model: | MixedLM | Dependent Variable: | clinresp |
| --- | --- | --- | --- |
| No. Observations: | 386 | Method: | REML |
| No. Groups: | 20 | Scale: | 0.0914 |
| Min. group size: | 2 | Log-Likelihood: | -123.8498 |
| Max. group size: | 43 | Converged: | Yes |
| Mean group size: | 19.3 |  |  |

|  | **Coef.** | **Std.Err.** | **z** | **P>\|z\|** | **[0.025** | **0.975]** |
| --- | --- | --- | --- | --- | --- | --- |
| **Intercept** | -0.437 | 0.092 | -4.765 | 0.000 | -0.617 | -0.257 |
| **C(pmnt)[T.1.0]** | -0.028 | 0.063 | -0.442 | 0.659 | -0.151 | 0.096 |
| **C(pmnt)[T.2.0]** | 0.181 | 0.051 | 3.578 | 0.000 | 0.082 | 0.281 |
| **C(sex)[T.M]** | 0.008 | 0.033 | 0.241 | 0.810 | -0.057 | 0.073 |
| **age** | -0.004 | 0.001 | -3.129 | 0.002 | -0.007 | -0.002 |
| **volpc1** | 0.000 | 0.003 | 0.035 | 0.972 | -0.006 | 0.006 |
| **volpc2** | -0.014 | 0.007 | -2.133 | **0.033** | -0.028 | -0.001 |
| **nECT** | 0.004 | 0.004 | 1.072 | 0.284 | -0.003 | 0.011 |
| **Group Var** | 0.015 | 0.025 |  |  |  |  |

ΔMADRS ~ PC1_EF_ + PC2_EF_ + age + nECT + sex + placement + site

| Model: | MixedLM | Dependent Variable: | clinresp |
| --- | --- | --- | --- |
| No. Observations: | 386 | Method: | REML |
| No. Groups: | 20 | Scale: | 0.0918 |
| Min. group size: | 2 | Log-Likelihood: | -125.1054 |
| Max. group size: | 43 | Converged: | Yes |
| Mean group size: | 19.3 |  |  |

|  | **Coef.** | **Std.Err.** | **z** | **P>\|z\|** | **[0.025** | **0.975]** |
| --- | --- | --- | --- | --- | --- | --- |
| **Intercept** | -0.477 | 0.096 | -4.980 | 0.000 | -0.665 | -0.289 |
| **C(pmnt)[T.1.0]** | -0.029 | 0.063 | -0.465 | 0.642 | -0.153 | 0.094 |
| **C(pmnt)[T.2.0]** | 0.175 | 0.050 | 3.468 | 0.001 | 0.076 | 0.274 |
| **C(sex)[T.M]** | 0.020 | 0.036 | 0.566 | 0.572 | -0.050 | 0.090 |
| **age** | -0.004 | 0.001 | -2.631 | 0.009 | -0.006 | -0.001 |
| **efpc1** | 0.004 | 0.003 | 1.618 | 0.106 | -0.001 | 0.010 |
| **efpc2** | -0.002 | 0.005 | -0.348 | 0.728 | -0.013 | 0.009 |
| **nECT** | 0.005 | 0.003 | 1.405 | 0.160 | -0.002 | 0.012 |
| **Group Var** | 0.015 | 0.025 |  |  |  |  |

ΔMADRS ~ PC1_EF_ + PC2_EF_ + PC1_ΔVOL_ + PC2_ΔVOL_ + age + nECT + sex + placement + site

| Model: | MixedLM | Dependent Variable: | clinresp |
| --- | --- | --- | --- |
| No. Observations: | 386 | Method: | REML |
| No. Groups: | 20 | Scale: | 0.0916 |
| Min. group size: | 2 | Log-Likelihood: | -132.1979 |
| Max. group size: | 43 | Converged: | Yes |
| Mean group size: | 19.3 |  |  |

|  | **Coef.** | **Std.Err.** | **z** | **P>\|z\|** | **[0.025** | **0.975]** |
| --- | --- | --- | --- | --- | --- | --- |
| **Intercept** | -0.476 | 0.096 | -4.962 | 0.000 | -0.664 | -0.288 |
| **C(pmnt)[T.1.0]** | -0.028 | 0.062 | -0.445 | 0.656 | -0.150 | 0.094 |
| **C(pmnt)[T.2.0]** | 0.182 | 0.051 | 3.583 | 0.000 | 0.082 | 0.281 |
| **C(sex)[T.M]** | 0.028 | 0.036 | 0.781 | 0.435 | -0.043 | 0.099 |
| **age** | -0.003 | 0.001 | -2.478 | 0.013 | -0.006 | -0.001 |
| **efpc1** | 0.004 | 0.003 | 1.367 | 0.172 | -0.002 | 0.009 |
| **efpc2** | -0.002 | 0.006 | -0.373 | 0.709 | -0.013 | 0.009 |
| **volpc1** | 0.000 | 0.003 | 0.065 | 0.948 | -0.005 | 0.006 |
| **volpc2** | -0.013 | 0.007 | -1.955 | **0.051** | -0.027 | 0.000 |
| **nECT** | 0.004 | 0.004 | 0.975 | 0.330 | -0.004 | 0.011 |
| **Group Var** | 0.014 | 0.024 |  |  |  |  |

Additional Site-Specific Analyses

1) Leaving out high-resolution site

Site 10 exhibited T1 images with notably higher resolution (0.5 mm isotropic) compared to all other sites. We examined whether this resolution disparity might have affected our results. Given that this site comprised 25 RUL and 3 MIX cases, we focused our analysis on the RUL cases on the rest of the sites. We obtained very similar structures; the first PC1_ΔVOL_ accounted for 43% of the variance, while PC2_ΔVOL_ covered 6%. Notably, PC2_ΔVOL_ displayed a spatial similarity to the CDN of r=0.54, consistent with our main analysis using the entire dataset (Supplementary Figure 10 A).

2) Leaving out low magnetic field sites (1.5 T)

Sites 3 and 17 featured significantly lower magnetic field strengths compared to the other sites. We explored whether this magnetic field difference would yield differing outcomes. Given that these two sites only had most of their patients in BT placement, we focused on BT placements. We observed highly similar structures, with PC1_ΔVOL_ accounting for 43% of the variance and PC2_ΔVOL_ for 6%. Importantly, the latter displayed a high degree of similarity to the CDN, with a correlation coefficient of r=0.53 (Supplementary Figure 10 B).

SUPPLEMENTARY FIGURES:


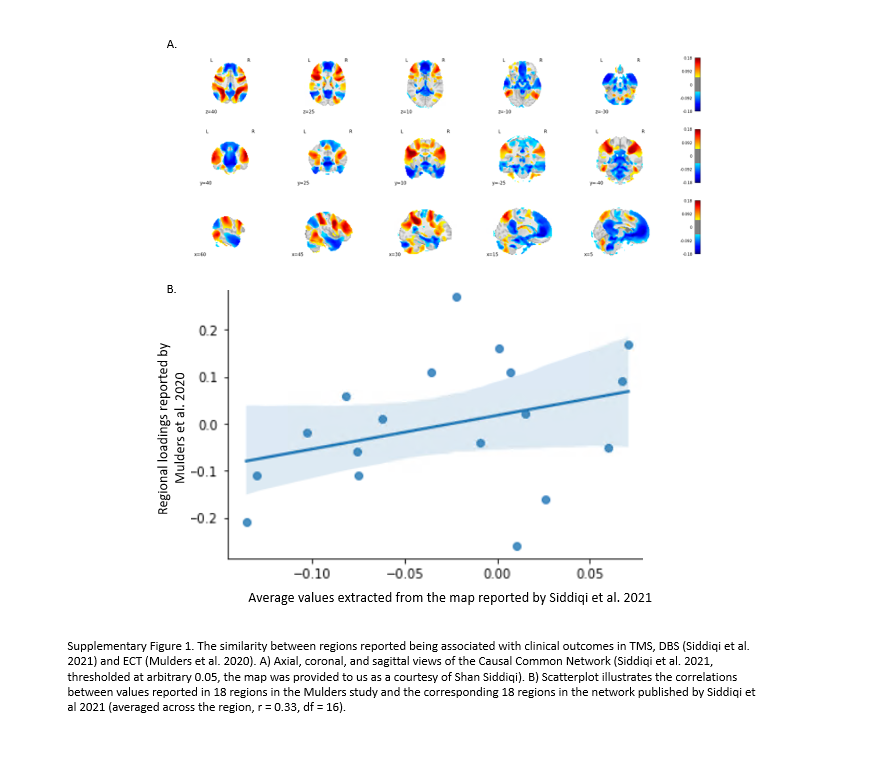


Supplementary Figure 1. The similarity between regions reported being associated with clinical outcomes in TMS, DBS (Siddiqi et al. 2021) and ECT (Mulders et al. 2020). A) Axial, coronal, and sagittal views of the Causal Common Network (Siddiqi et al. 2021, thresholded at arbitrary 0.05, the map was provided to us as a courtesy of Shan Siddiqi). B) Scatterplot illustrates the correlations between values reported in 18 regions in the Mulders study and the corresponding 18 regions in the network published by Siddiqi et al 2021 (averaged across the region, r = 0.33, df = 16).


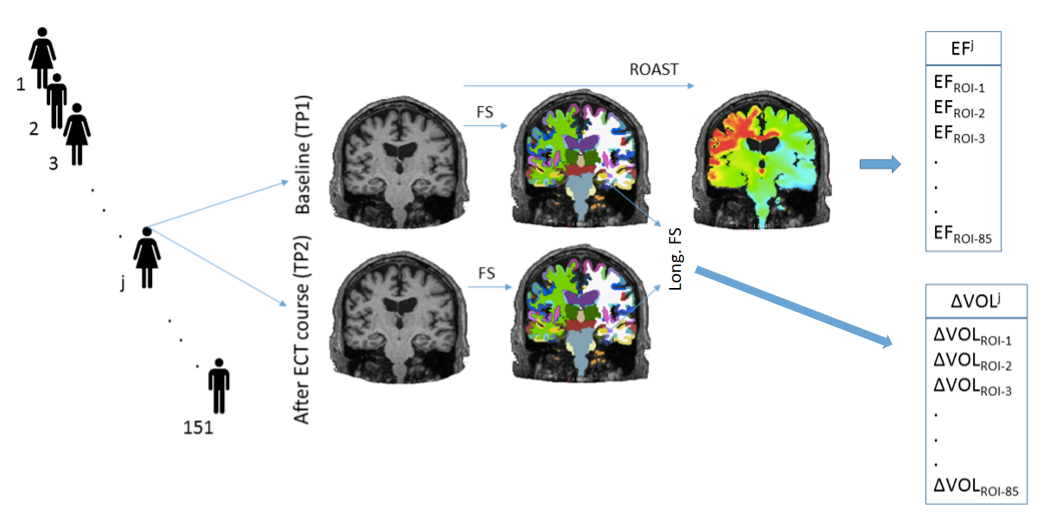


Supplementary Figure 2. Study design. Main data sources. 1) EF modeling with Roast based on the T1 baseline MRI. EF average is calculated for each segmented region. 2) Volume changes based on Freesurfer segmentation in 85 brain regions.


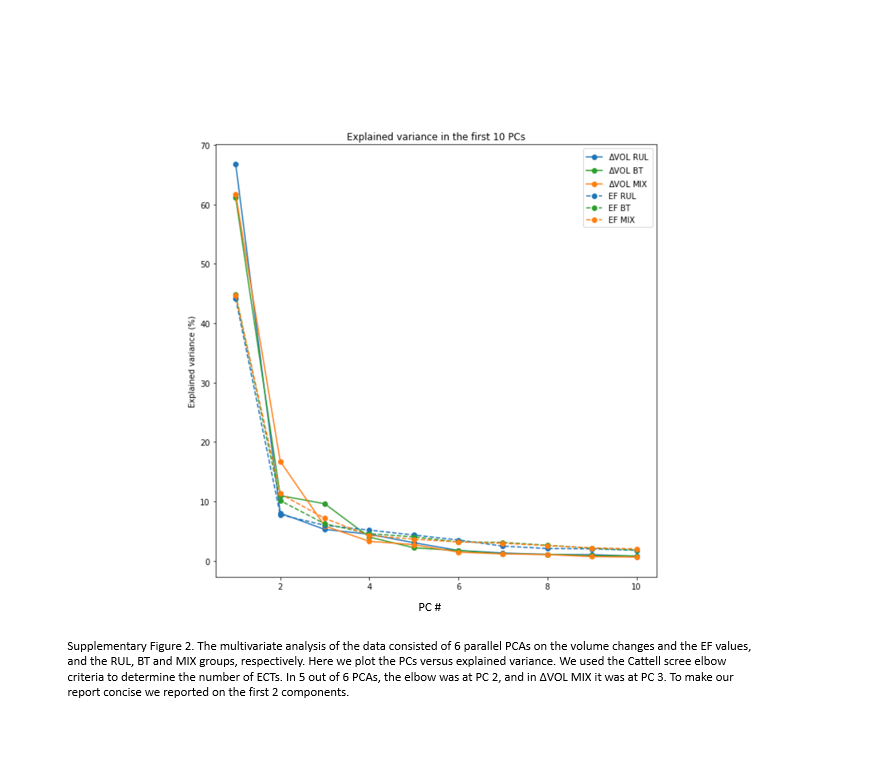


Supplementary Figure 3. The multivariate analysis of the data consisted of 6 parallel PCAs on the volume changes and the EF values, and the RUL, BT and MIX groups, respectively. Here we plot the PCs versus explained variance. We used the Cattell scree elbow criteria to determine the number of ECTs. In 5 out of 6 PCAs, the elbow was at PC 2, and in ΔVOL MIX it was at PC 3. To make our report concise we reported on the first 2 components.


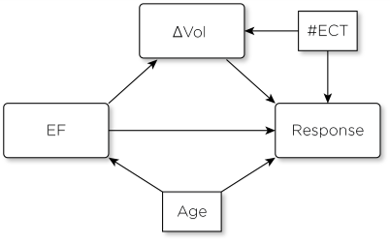


Supplementary Figure 4. In our multivariable regression analyses, we corrected for the number of ECT and for age as well. The rationale for this is illustrated on this causal model, with a directed acyclic graph (DAG). Age was chosen as a variable we controlled for as it was known to correlate with both clinical response and EF in the brain. When using ΔVOL as a covariate, it can open a spurious correlation between EF and nECT and in this way between EF and clinical response.


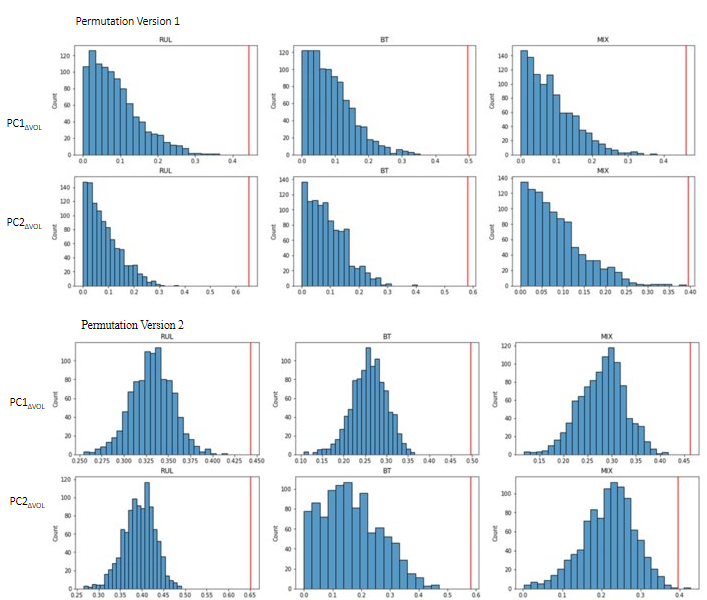


Supplementary Figure 5. Permutation tests. The top panel represents the initial permutation iteration (see supplementary methods), while the bottom panel depicts the more conservative permutation version (applicable only in ΔVOL, not in EF). The red lines signify the "real" correlation values between our Principal Components (PCs) and the DCN. The histogram displays the distribution of correlation values across permutations. These findings collectively suggest that the observed correlation greatly exceeds what would be anticipated from random sampling (p<0.001).


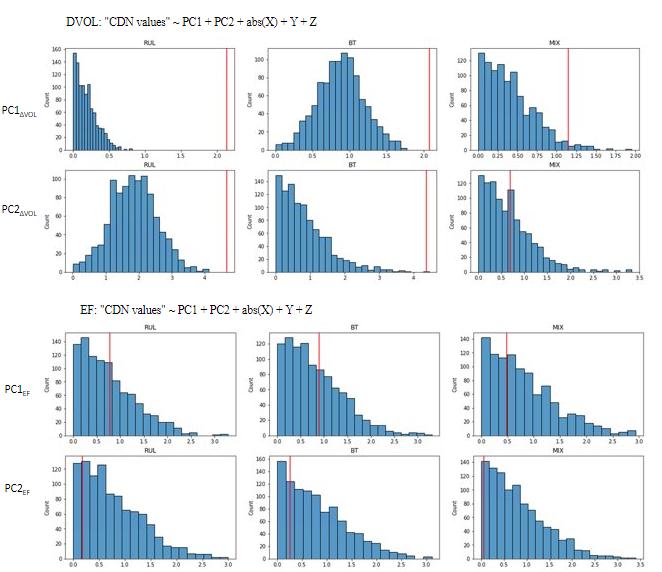


Supplementary Figure 6. Permutation tests to support that volumetric principal components are more similar than expected just based on x, y, and z coordinates. Also, the lower panel shows that this is not true for EF principal components.


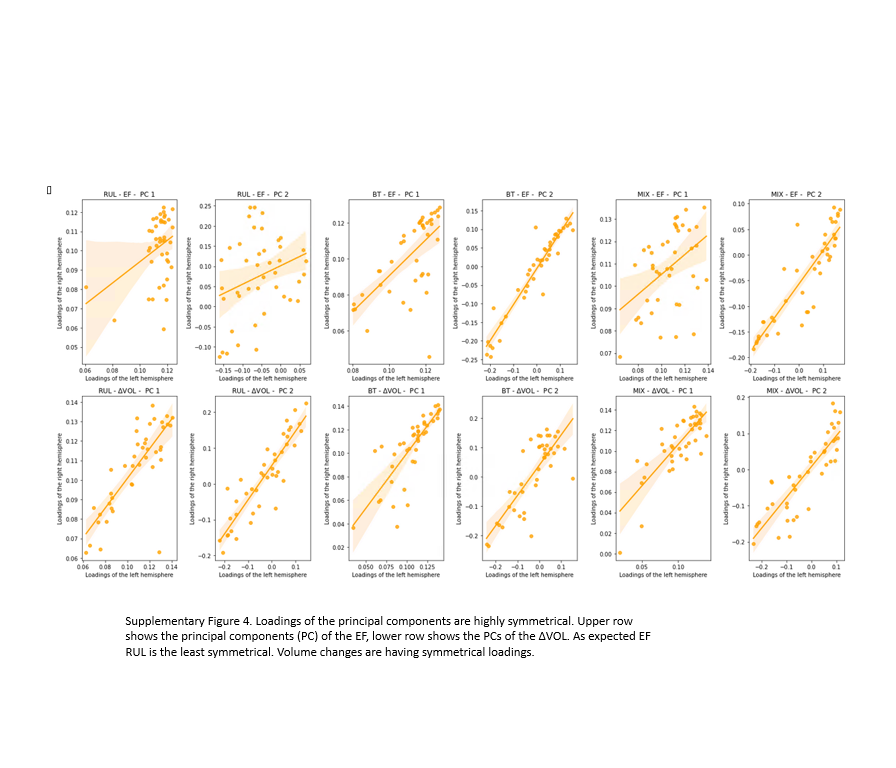


Supplementary Figure 7. Loadings of the principal components are highly symmetrical. Upper row shows the principal components (PC) of the EF, lower row shows the PCs of the ΔVOL. As expected EF RUL is the least symmetrical. Volume changes are having symmetrical loadings.


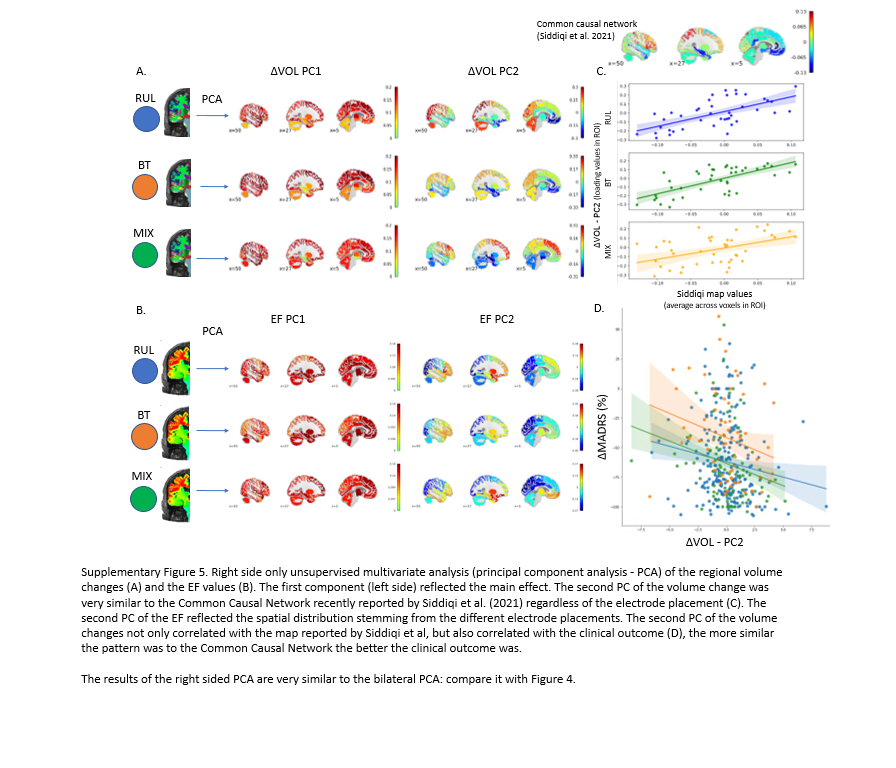


Supplementary Figure 8. Right side only unsupervised multivariate analysis (principal component analysis - PCA) of the regional volume changes (A) and the EF values (B). The first component (left side) reflected the main effect. The second PC of the volume change was very similar to the Common Causal Network recently reported by Siddiqi et al. (2021) regardless of the electrode placement (C). The second PC of the EF reflected the spatial distribution stemming from the different electrode placements. The second PC of the volume changes not only correlated with the map reported by Siddiqi et al, but also correlated with the clinical outcome (D), the more similar the pattern was to the Common Causal Network the better the clinical outcome was.

The results of the right sided PCA are very similar to the bilateral PCA: compare it with Figure 4.


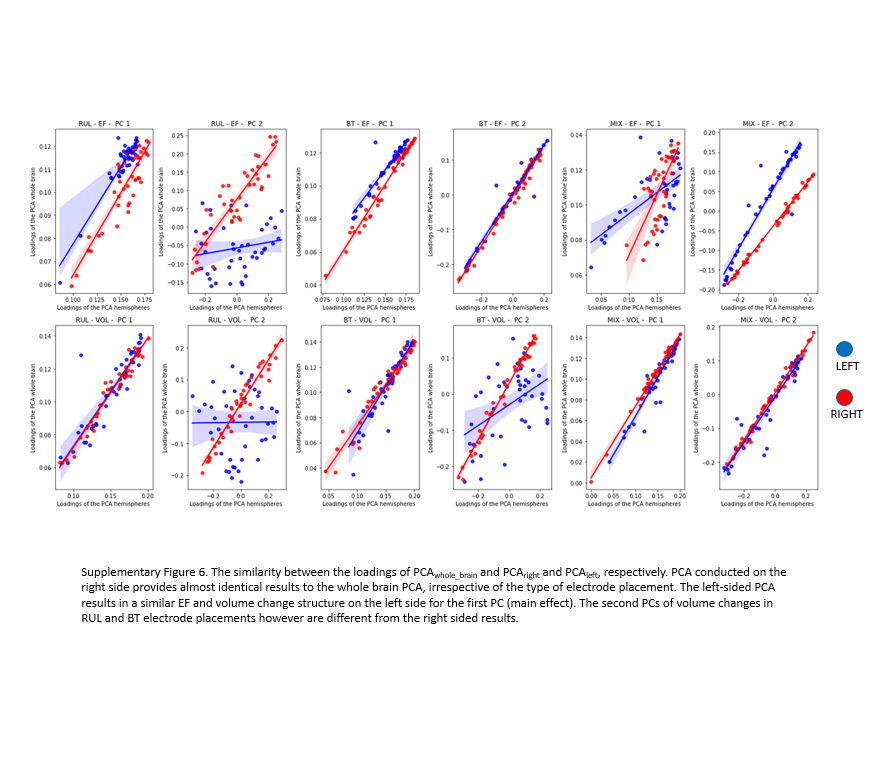


Supplementary Figure 9. The similarity between the loadings of PCA_whole_brain_ and PCA_right_ and PCA_left_, respectively. PCA conducted on the right side provides almost identical results to the whole brain PCA, irrespective of the type of electrode placement. The left-sided PCA results in a similar EF and volume change structure on the left side for the first PC (main effect). The second PCs of volume changes in RUL and BT electrode placements however are different from the right sided results.


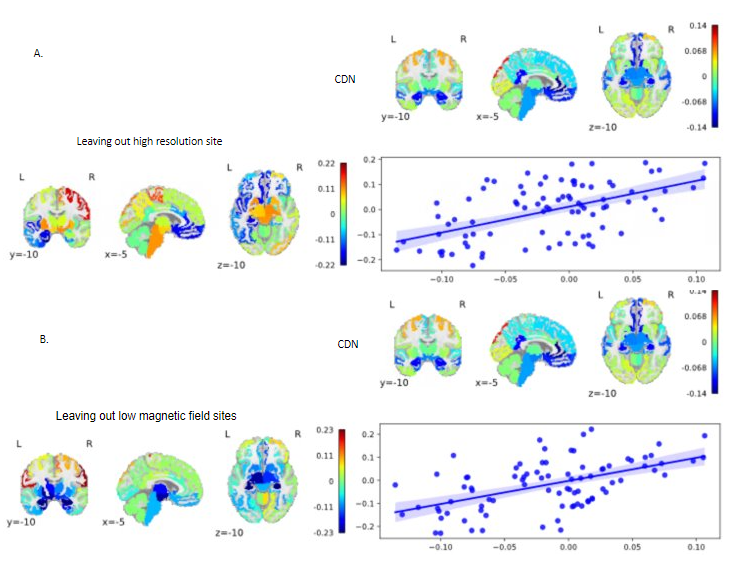


Supplementary Figure 10.

SUPPLEMENTARY TABLES:

1. A.) Demographics:

|  | N | Age (sd) | Medications (med. free, SSRI/SNRI, TCA, MAOI, AP*) | Average  number  of ECT | Baseline  MADRS | ΔMADRS  (%) |
| --- | --- | --- | --- | --- | --- | --- |
| All | 386 | 54.4 (15.6) | (126, 143, 60, 1, 139) | 12.5 | 25.5 | -59.0 |
| Female | 233 | 54.8 (16.1) | (74, 83, 44, 0, 90) | 12.5 | 25.9 | -58.9 |
| Male | 153 | 53.9 (15.0) | (52, 60, 16, 1, 49) | 12.4 | 25.0 | -59.2 |

*med. free: medication free, SSRI: selective serotonin reuptake inhibitor, SNRI: serotonin and norepinephrine reuptake inhibitor, TCA: tricyclic antidepressant, MAOI: monoamine oxidase inhibitors, AP: antipsychotic medications

B.) Sample sizes in 19 sites from the GEMRIC consortium:

| Site | RUL | BT | MIX | N | Field (T) | Resolution (mm) |
| --- | --- | --- | --- | --- | --- | --- |
| 1 | 30 | 1 | 12 | 43 | 3 | 1.0x1.0x1.3 |
| 2 | 5 | 1 | 2 | 8 | 3 | 1.05x1.05x1.2 |
| 3 | 7 | 3 | 13 | 23 | 1.5 | 1.1x1.1x1.1 |
| 4 | 12 | 0 | 3 | 15 | 3 | 1.0x1.0x0.8 |
| 5 | 33 | 2 | 4 | 39 | 3 | 1.0x1.0x1.0 |
| 6 | 15 | 2 | 13 | 30 | 3 | 1.0x1.0x1.0 |
| 7 | 15 | 0 | 0 | 15 | 3 | 1.0x1.0x1.0 |
| 8 | 16 | 0 | 0 | 16 | 3 | 1.0x1.0x1.0 |
| 9 | 0 | 14 | 0 | 14 | 3 | 1.0x1.0x1.0 |
| 10 | 25 | 0 | 3 | 28 | 3 | 0.5x0.5x0.5 |
| 11 | 10 | 0 | 0 | 10 | 3 | 1.0x1.0x1.0 |
| 12 | 10 | 0 | 2 | 12 | 3 | 1.0x1.0x1.0 |
| 13 | 2 | 0 | 0 | 2 | 3 | 1.0x1.0x1.0 |
| 14 | 30 | 0 | 0 | 30 | 3 | 1.0x1.0x1.0 |
| 15 | 0 | 10 | 0 | 10 | 3 | 0.94x0.94x1.0 |
| 16 | 12 | 1 | 2 | 15 | 3 | 1.0x1.0x1.0 |
| 17 | 0 | 19 | 0 | 19 | 1.5 | 1.0x1.0x1.0 |
| 18 | 0 | 11 | 0 | 11 | 3 | 1.0x1.0x1.0 |
| 19 | 24 | 0 | 5 | 29 | 3 | 1.0x1.0x1.0 |
| 20 | 0 | 15 | 2 | 17 | 3 | 1.0x1.0x1.0 |
| Sum | 246 | 79 | 61 | 386 |  |  |

2. A) Averaged electric field and volume changes across all 85 regions:

| ROI | EF  (V/m) | Volume  change (%) | Volume change  (Cohen’s d) |
| --- | --- | --- | --- |
| Brain_Stem | 58.30 | 0.09 | 0.10 |
| Left_Accumbens_area | 85.71 | 1.40 | 0.59 |
| Left_Amygdala | 66.09 | 4.18 | 1.18 |
| Left_Caudate | 56.07 | 1.34 | 0.80 |
| Left_Cerebellum_Cortex | 36.23 | -0.02 | -0.02 |
| Left_Hippocampus | 60.87 | 2.31 | 1.12 |
| Left_Pallidum | 86.12 | 0.97 | 0.87 |
| Left_Putamen | 80.46 | 1.05 | 0.93 |
| Left_Thalamus | 61.69 | 1.04 | 0.94 |
| Left_VentralDC | 76.93 | 0.49 | 0.53 |
| Right_Accumbens_area | 114.91 | 2.20 | 1.00 |
| Right_Amygdala | 91.77 | 6.77 | 1.93 |
| Right_Caudate | 106.78 | 1.47 | 0.89 |
| Right_Cerebellum_Cortex | 39.99 | 0.03 | 0.03 |
| Right_Hippocampus | 85.22 | 2.89 | 1.61 |
| Right_Pallidum | 128.32 | 1.15 | 1.04 |
| Right_Putamen | 129.47 | 1.27 | 1.04 |
| Right_Thalamus | 86.98 | 1.35 | 1.21 |
| Right_VentralDC | 93.97 | 0.62 | 0.69 |
| ctx_lh_caudalanteriorcingulate | 64.29 | 1.90 | 1.02 |
| ctx_lh_caudalmiddlefrontal | 67.68 | 1.00 | 0.63 |
| ctx_lh_cuneus | 46.00 | 0.46 | 0.41 |
| ctx_lh_entorhinal | 53.66 | 2.66 | 0.86 |
| ctx_lh_frontalpole | 57.15 | 0.66 | 0.22 |
| ctx_lh_fusiform | 46.47 | 1.24 | 0.88 |
| ctx_lh_inferiorparietal | 44.22 | 1.01 | 0.65 |
| ctx_lh_inferiortemporal | 53.48 | 1.24 | 0.70 |
| ctx_lh_insula | 65.70 | 1.77 | 1.04 |
| ctx_lh_isthmuscingulate | 63.37 | 0.24 | 0.18 |
| ctx_lh_lateraloccipital | 39.73 | 0.71 | 0.52 |
| ctx_lh_lateralorbitofrontal | 68.54 | 0.61 | 0.37 |
| ctx_lh_lingual | 39.57 | 0.51 | 0.51 |
| ctx_lh_medialorbitofrontal | 57.71 | 0.70 | 0.41 |
| ctx_lh_middletemporal | 54.36 | 1.05 | 0.54 |
| ctx_lh_paracentral | 57.86 | 1.13 | 0.79 |
| ctx_lh_parahippocampal | 51.86 | 1.60 | 0.79 |
| ctx_lh_parsopercularis | 64.32 | 1.28 | 0.85 |
| ctx_lh_parsorbitalis | 60.72 | 0.74 | 0.33 |
| ctx_lh_parstriangularis | 61.99 | 1.01 | 0.59 |
| ctx_lh_pericalcarine | 40.59 | 0.44 | 0.41 |
| ctx_lh_postcentral | 55.88 | 0.61 | 0.52 |
| ctx_lh_posteriorcingulate | 76.31 | 0.59 | 0.44 |
| ctx_lh_precentral | 61.27 | 0.99 | 0.80 |
| ctx_lh_precuneus | 53.23 | 0.53 | 0.45 |
| ctx_lh_rostralanteriorcingulate | 52.90 | 1.20 | 0.59 |
| ctx_lh_rostralmiddlefrontal | 62.56 | 0.77 | 0.48 |
| ctx_lh_superiorfrontal | 65.29 | 1.10 | 0.71 |
| ctx_lh_superiorparietal | 45.80 | 0.76 | 0.53 |
| ctx_lh_superiortemporal | 55.54 | 1.16 | 0.67 |
| ctx_lh_supramarginal | 52.50 | 1.09 | 0.73 |
| ctx_lh_temporalpole | 52.71 | 2.97 | 0.85 |
| ctx_lh_transversetemporal | 50.60 | 0.81 | 0.48 |
| ctx_rh_caudalanteriorcingulate | 74.92 | 1.90 | 1.12 |
| ctx_rh_caudalmiddlefrontal | 146.91 | 1.11 | 0.66 |
| ctx_rh_cuneus | 50.68 | 0.58 | 0.55 |
| ctx_rh_entorhinal | 88.96 | 3.57 | 1.27 |
| ctx_rh_frontalpole | 79.97 | 1.02 | 0.35 |
| ctx_rh_fusiform | 73.68 | 1.77 | 1.25 |
| ctx_rh_inferiorparietal | 82.94 | 1.17 | 0.77 |
| ctx_rh_inferiortemporal | 98.21 | 1.91 | 1.12 |
| ctx_rh_insula | 108.00 | 2.69 | 1.38 |
| ctx_rh_isthmuscingulate | 70.83 | 0.28 | 0.23 |
| ctx_rh_lateraloccipital | 52.49 | 0.88 | 0.63 |
| ctx_rh_lateralorbitofrontal | 110.93 | 1.01 | 0.58 |
| ctx_rh_lingual | 47.19 | 0.67 | 0.69 |
| ctx_rh_medialorbitofrontal | 87.66 | 1.19 | 0.69 |
| ctx_rh_middletemporal | 137.53 | 1.58 | 0.89 |
| ctx_rh_paracentral | 84.12 | 1.22 | 0.92 |
| ctx_rh_parahippocampal | 74.61 | 2.27 | 1.12 |
| ctx_rh_parsopercularis | 144.00 | 1.27 | 0.81 |
| ctx_rh_parsorbitalis | 139.34 | 1.04 | 0.46 |
| ctx_rh_parstriangularis | 174.54 | 1.23 | 0.75 |
| ctx_rh_pericalcarine | 46.90 | 0.53 | 0.53 |
| ctx_rh_postcentral | 144.15 | 0.70 | 0.56 |
| ctx_rh_posteriorcingulate | 84.51 | 0.70 | 0.55 |
| ctx_rh_precentral | 146.31 | 1.03 | 0.76 |
| ctx_rh_precuneus | 63.72 | 0.62 | 0.59 |
| ctx_rh_rostralanteriorcingulate | 66.46 | 1.99 | 0.97 |
| ctx_rh_rostralmiddlefrontal | 114.64 | 0.98 | 0.58 |
| ctx_rh_superiorfrontal | 86.64 | 1.19 | 0.76 |
| ctx_rh_superiorparietal | 66.77 | 0.76 | 0.53 |
| ctx_rh_superiortemporal | 137.87 | 2.05 | 1.21 |
| ctx_rh_supramarginal | 126.66 | 1.30 | 0.82 |
| ctx_rh_temporalpole | 102.41 | 4.63 | 1.39 |
| ctx_rh_transversetemporal | 102.67 | 1.45 | 0.80 |

2 B.) Averaged electric field and volume changes **in RUL only** patients across all 85 regions:

| ROI | EF  (V/m) | Volume  change (%) | Volume change  (Cohen’s d) |
| --- | --- | --- | --- |
| Brain_Stem | 56.94 | 0.07 | 0.08 |
| Left_Accumbens_area | 78.28 | 1.01 | 0.46 |
| Left_Amygdala | 45.40 | 2.84 | 1.01 |
| Left_Caudate | 42.57 | 1.17 | 0.74 |
| Left_Cerebellum_Cortex | 36.49 | -0.02 | -0.02 |
| Left_Hippocampus | 49.00 | 1.66 | 0.87 |
| Left_Pallidum | 62.53 | 0.82 | 0.74 |
| Left_Putamen | 51.80 | 0.93 | 0.82 |
| Left_Thalamus | 57.97 | 0.94 | 0.86 |
| Left_VentralDC | 75.71 | 0.42 | 0.49 |
| Right_Accumbens_area | 114.25 | 2.05 | 0.90 |
| Right_Amygdala | 79.66 | 6.27 | 1.85 |
| Right_Caudate | 98.14 | 1.32 | 0.80 |
| Right_Cerebellum_Cortex | 41.36 | 0.00 | 0.00 |
| Right_Hippocampus | 81.16 | 2.67 | 1.46 |
| Right_Pallidum | 119.18 | 1.10 | 0.99 |
| Right_Putamen | 117.99 | 1.23 | 0.99 |
| Right_Thalamus | 87.25 | 1.29 | 1.14 |
| Right_VentralDC | 96.14 | 0.54 | 0.61 |
| ctx_lh_caudalanteriorcingulate | 75.88 | 1.84 | 0.98 |
| ctx_lh_caudalmiddlefrontal | 58.46 | 0.95 | 0.62 |
| ctx_lh_cuneus | 56.33 | 0.56 | 0.52 |
| ctx_lh_entorhinal | 33.82 | 1.97 | 0.67 |
| ctx_lh_frontalpole | 38.83 | 0.52 | 0.17 |
| ctx_lh_fusiform | 37.97 | 1.11 | 0.79 |
| ctx_lh_inferiorparietal | 45.12 | 0.98 | 0.64 |
| ctx_lh_inferiortemporal | 35.43 | 1.07 | 0.62 |
| ctx_lh_insula | 42.11 | 1.56 | 0.91 |
| ctx_lh_isthmuscingulate | 76.55 | 0.31 | 0.23 |
| ctx_lh_lateraloccipital | 42.69 | 0.72 | 0.54 |
| ctx_lh_lateralorbitofrontal | 39.74 | 0.39 | 0.24 |
| ctx_lh_lingual | 41.92 | 0.53 | 0.51 |
| ctx_lh_medialorbitofrontal | 45.82 | 0.41 | 0.26 |
| ctx_lh_middletemporal | 36.58 | 0.80 | 0.42 |
| ctx_lh_paracentral | 67.94 | 1.18 | 0.83 |
| ctx_lh_parahippocampal | 46.16 | 1.50 | 0.73 |
| ctx_lh_parsopercularis | 45.68 | 1.20 | 0.81 |
| ctx_lh_parsorbitalis | 33.83 | 0.49 | 0.22 |
| ctx_lh_parstriangularis | 36.98 | 0.84 | 0.52 |
| ctx_lh_pericalcarine | 46.63 | 0.49 | 0.47 |
| ctx_lh_postcentral | 50.78 | 0.67 | 0.57 |
| ctx_lh_posteriorcingulate | 95.60 | 0.71 | 0.52 |
| ctx_lh_precentral | 54.39 | 0.95 | 0.76 |
| ctx_lh_precuneus | 63.56 | 0.65 | 0.55 |
| ctx_lh_rostralanteriorcingulate | 54.80 | 0.95 | 0.48 |
| ctx_lh_rostralmiddlefrontal | 43.21 | 0.64 | 0.43 |
| ctx_lh_superiorfrontal | 65.70 | 1.03 | 0.68 |
| ctx_lh_superiorparietal | 48.22 | 0.81 | 0.58 |
| ctx_lh_superiortemporal | 37.03 | 0.83 | 0.50 |
| ctx_lh_supramarginal | 48.23 | 1.10 | 0.75 |
| ctx_lh_temporalpole | 28.07 | 2.16 | 0.67 |
| ctx_lh_transversetemporal | 38.73 | 0.67 | 0.38 |
| ctx_rh_caudalanteriorcingulate | 85.53 | 1.92 | 1.16 |
| ctx_rh_caudalmiddlefrontal | 168.80 | 1.03 | 0.62 |
| ctx_rh_cuneus | 61.18 | 0.65 | 0.61 |
| ctx_rh_entorhinal | 80.96 | 3.37 | 1.21 |
| ctx_rh_frontalpole | 69.28 | 0.96 | 0.31 |
| ctx_rh_fusiform | 73.06 | 1.81 | 1.24 |
| ctx_rh_inferiorparietal | 95.61 | 1.12 | 0.71 |
| ctx_rh_inferiortemporal | 93.84 | 1.88 | 1.14 |
| ctx_rh_insula | 107.72 | 2.69 | 1.36 |
| ctx_rh_isthmuscingulate | 83.21 | 0.31 | 0.26 |
| ctx_rh_lateraloccipital | 59.10 | 0.93 | 0.64 |
| ctx_rh_lateralorbitofrontal | 100.94 | 0.91 | 0.50 |
| ctx_rh_lingual | 51.29 | 0.72 | 0.73 |
| ctx_rh_medialorbitofrontal | 80.33 | 1.13 | 0.63 |
| ctx_rh_middletemporal | 139.81 | 1.51 | 0.87 |
| ctx_rh_paracentral | 99.20 | 1.24 | 0.95 |
| ctx_rh_parahippocampal | 74.85 | 2.34 | 1.10 |
| ctx_rh_parsopercularis | 153.76 | 1.14 | 0.72 |
| ctx_rh_parsorbitalis | 133.34 | 0.82 | 0.38 |
| ctx_rh_parstriangularis | 178.76 | 1.15 | 0.70 |
| ctx_rh_pericalcarine | 54.18 | 0.55 | 0.54 |
| ctx_rh_postcentral | 170.38 | 0.74 | 0.62 |
| ctx_rh_posteriorcingulate | 104.03 | 0.82 | 0.64 |
| ctx_rh_precentral | 170.67 | 0.96 | 0.71 |
| ctx_rh_precuneus | 75.78 | 0.71 | 0.66 |
| ctx_rh_rostralanteriorcingulate | 66.41 | 1.84 | 1.01 |
| ctx_rh_rostralmiddlefrontal | 115.08 | 0.92 | 0.57 |
| ctx_rh_superiorfrontal | 92.38 | 1.12 | 0.74 |
| ctx_rh_superiorparietal | 78.11 | 0.77 | 0.53 |
| ctx_rh_superiortemporal | 142.93 | 2.05 | 1.24 |
| ctx_rh_supramarginal | 147.28 | 1.35 | 0.88 |
| ctx_rh_temporalpole | 96.09 | 4.22 | 1.29 |
| ctx_rh_transversetemporal | 110.55 | 1.50 | 0.80 |

2 C.) Averaged electric field and volume changes **in BT only** patients across all 85 regions:

| ROI | EF  (V/m) | Volume  change (%) | Volume change  (Cohen’s d) |
| --- | --- | --- | --- |
| Brain_Stem | 62.84 | 0.17 | 0.21 |
| Left_Accumbens_area | 106.16 | 2.26 | 1.03 |
| Left_Amygdala | 119.30 | 6.88 | 2.05 |
| Left_Caudate | 90.28 | 1.63 | 0.84 |
| Left_Cerebellum_Cortex | 36.30 | -0.08 | -0.10 |
| Left_Hippocampus | 91.83 | 3.47 | 2.09 |
| Left_Pallidum | 147.27 | 1.20 | 1.25 |
| Left_Putamen | 154.02 | 1.28 | 1.25 |
| Left_Thalamus | 72.28 | 1.25 | 1.10 |
| Left_VentralDC | 81.54 | 0.56 | 0.54 |
| Right_Accumbens_area | 117.71 | 2.38 | 1.16 |
| Right_Amygdala | 123.52 | 7.12 | 2.26 |
| Right_Caudate | 127.81 | 1.72 | 1.04 |
| Right_Cerebellum_Cortex | 37.40 | 0.17 | 0.17 |
| Right_Hippocampus | 96.67 | 3.10 | 2.01 |
| Right_Pallidum | 152.28 | 1.18 | 1.08 |
| Right_Putamen | 159.12 | 1.15 | 1.04 |
| Right_Thalamus | 87.10 | 1.36 | 1.22 |
| Right_VentralDC | 89.77 | 0.82 | 0.83 |
| ctx_lh_caudalanteriorcingulate | 36.16 | 2.04 | 1.05 |
| ctx_lh_caudalmiddlefrontal | 93.05 | 1.15 | 0.61 |
| ctx_lh_cuneus | 20.97 | 0.35 | 0.38 |
| ctx_lh_entorhinal | 104.43 | 4.03 | 1.30 |
| ctx_lh_frontalpole | 105.16 | 1.05 | 0.36 |
| ctx_lh_fusiform | 69.06 | 1.51 | 1.08 |
| ctx_lh_inferiorparietal | 43.25 | 0.91 | 0.53 |
| ctx_lh_inferiortemporal | 100.33 | 1.49 | 0.83 |
| ctx_lh_insula | 126.11 | 2.31 | 1.38 |
| ctx_lh_isthmuscingulate | 31.64 | 0.09 | 0.09 |
| ctx_lh_lateraloccipital | 33.33 | 0.72 | 0.56 |
| ctx_lh_lateralorbitofrontal | 142.06 | 1.12 | 0.62 |
| ctx_lh_lingual | 34.60 | 0.51 | 0.60 |
| ctx_lh_medialorbitofrontal | 87.96 | 1.27 | 0.65 |
| ctx_lh_middletemporal | 100.25 | 1.44 | 0.83 |
| ctx_lh_paracentral | 33.43 | 1.01 | 0.67 |
| ctx_lh_parahippocampal | 67.17 | 1.98 | 1.02 |
| ctx_lh_parsopercularis | 111.32 | 1.50 | 0.94 |
| ctx_lh_parsorbitalis | 129.41 | 1.41 | 0.64 |
| ctx_lh_parstriangularis | 125.17 | 1.49 | 0.77 |
| ctx_lh_pericalcarine | 26.33 | 0.40 | 0.40 |
| ctx_lh_postcentral | 70.10 | 0.41 | 0.33 |
| ctx_lh_posteriorcingulate | 29.60 | 0.32 | 0.23 |
| ctx_lh_precentral | 79.88 | 1.10 | 0.81 |
| ctx_lh_precuneus | 28.51 | 0.26 | 0.25 |
| ctx_lh_rostralanteriorcingulate | 48.54 | 1.78 | 0.76 |
| ctx_lh_rostralmiddlefrontal | 112.82 | 1.16 | 0.61 |
| ctx_lh_superiorfrontal | 66.10 | 1.24 | 0.71 |
| ctx_lh_superiorparietal | 41.12 | 0.65 | 0.43 |
| ctx_lh_superiortemporal | 103.46 | 1.78 | 1.06 |
| ctx_lh_supramarginal | 64.55 | 0.91 | 0.63 |
| ctx_lh_temporalpole | 115.74 | 4.72 | 1.31 |
| ctx_lh_transversetemporal | 81.27 | 1.03 | 0.67 |
| ctx_rh_caudalanteriorcingulate | 49.46 | 1.93 | 1.08 |
| ctx_rh_caudalmiddlefrontal | 96.61 | 1.36 | 0.79 |
| ctx_rh_cuneus | 25.12 | 0.45 | 0.45 |
| ctx_rh_entorhinal | 110.58 | 3.92 | 1.33 |
| ctx_rh_frontalpole | 108.34 | 1.29 | 0.45 |
| ctx_rh_fusiform | 76.91 | 1.72 | 1.32 |
| ctx_rh_inferiorparietal | 52.68 | 1.28 | 0.86 |
| ctx_rh_inferiortemporal | 110.70 | 1.93 | 1.08 |
| ctx_rh_insula | 109.91 | 2.63 | 1.43 |
| ctx_rh_isthmuscingulate | 40.68 | 0.35 | 0.30 |
| ctx_rh_lateraloccipital | 37.09 | 0.85 | 0.66 |
| ctx_rh_lateralorbitofrontal | 137.08 | 1.19 | 0.73 |
| ctx_rh_lingual | 37.84 | 0.63 | 0.71 |
| ctx_rh_medialorbitofrontal | 107.39 | 1.21 | 0.76 |
| ctx_rh_middletemporal | 131.87 | 1.75 | 0.89 |
| ctx_rh_paracentral | 47.09 | 1.26 | 0.92 |
| ctx_rh_parahippocampal | 74.91 | 2.15 | 1.17 |
| ctx_rh_parsopercularis | 120.84 | 1.70 | 1.13 |
| ctx_rh_parsorbitalis | 156.31 | 1.52 | 0.67 |
| ctx_rh_parstriangularis | 165.73 | 1.33 | 0.82 |
| ctx_rh_pericalcarine | 29.50 | 0.54 | 0.56 |
| ctx_rh_postcentral | 79.69 | 0.68 | 0.47 |
| ctx_rh_posteriorcingulate | 37.28 | 0.48 | 0.36 |
| ctx_rh_precentral | 87.22 | 1.25 | 0.91 |
| ctx_rh_precuneus | 34.68 | 0.51 | 0.49 |
| ctx_rh_rostralanteriorcingulate | 67.55 | 2.20 | 0.97 |
| ctx_rh_rostralmiddlefrontal | 116.09 | 1.16 | 0.67 |
| ctx_rh_superiorfrontal | 74.34 | 1.35 | 0.79 |
| ctx_rh_superiorparietal | 39.81 | 0.81 | 0.56 |
| ctx_rh_superiortemporal | 128.38 | 2.00 | 1.19 |
| ctx_rh_supramarginal | 75.81 | 1.19 | 0.74 |
| ctx_rh_temporalpole | 120.51 | 5.41 | 1.52 |
| ctx_rh_transversetemporal | 84.19 | 1.31 | 0.79 |

2 D.) Averaged electric field and volume changes i**n MIX** patients across all 85 regions:

| ROI | EF  (V/m) | Volume  change (%) | Volume change  (Cohen’s d) |
| --- | --- | --- | --- |
| Brain_Stem | 57.93 | 0.05 | 0.05 |
| Left_Accumbens_area | 89.19 | 1.84 | 0.65 |
| Left_Amygdala | 80.62 | 6.12 | 1.73 |
| Left_Caudate | 66.18 | 1.64 | 1.06 |
| Left_Cerebellum_Cortex | 35.10 | 0.08 | 0.06 |
| Left_Hippocampus | 68.69 | 3.42 | 1.65 |
| Left_Pallidum | 102.04 | 1.30 | 1.05 |
| Left_Putamen | 100.76 | 1.25 | 1.04 |
| Left_Thalamus | 62.98 | 1.19 | 1.11 |
| Left_VentralDC | 75.89 | 0.66 | 0.69 |
| Right_Accumbens_area | 113.95 | 2.56 | 1.23 |
| Right_Amygdala | 99.51 | 8.37 | 2.14 |
| Right_Caudate | 114.41 | 1.74 | 1.08 |
| Right_Cerebellum_Cortex | 37.79 | -0.02 | -0.02 |
| Right_Hippocampus | 86.76 | 3.50 | 1.94 |
| Right_Pallidum | 134.17 | 1.33 | 1.17 |
| Right_Putamen | 137.36 | 1.57 | 1.26 |
| Right_Thalamus | 85.75 | 1.59 | 1.47 |
| Right_VentralDC | 90.62 | 0.69 | 0.86 |
| ctx_lh_caudalanteriorcingulate | 53.95 | 1.96 | 1.21 |
| ctx_lh_caudalmiddlefrontal | 72.03 | 0.99 | 0.71 |
| ctx_lh_cuneus | 36.73 | 0.17 | 0.13 |
| ctx_lh_entorhinal | 67.92 | 3.67 | 1.37 |
| ctx_lh_frontalpole | 68.81 | 0.69 | 0.26 |
| ctx_lh_fusiform | 51.47 | 1.41 | 1.03 |
| ctx_lh_inferiorparietal | 41.85 | 1.27 | 0.81 |
| ctx_lh_inferiortemporal | 65.62 | 1.58 | 0.89 |
| ctx_lh_insula | 82.58 | 1.89 | 1.22 |
| ctx_lh_isthmuscingulate | 51.31 | 0.17 | 0.10 |
| ctx_lh_lateraloccipital | 36.07 | 0.67 | 0.40 |
| ctx_lh_lateralorbitofrontal | 89.44 | 0.87 | 0.59 |
| ctx_lh_lingual | 36.51 | 0.42 | 0.41 |
| ctx_lh_medialorbitofrontal | 66.51 | 1.16 | 0.69 |
| ctx_lh_middletemporal | 66.62 | 1.56 | 0.69 |
| ctx_lh_paracentral | 48.82 | 1.10 | 0.83 |
| ctx_lh_parahippocampal | 55.07 | 1.53 | 0.74 |
| ctx_lh_parsopercularis | 78.63 | 1.32 | 0.91 |
| ctx_lh_parsorbitalis | 80.25 | 0.91 | 0.40 |
| ctx_lh_parstriangularis | 81.03 | 1.08 | 0.64 |
| ctx_lh_pericalcarine | 34.68 | 0.30 | 0.24 |
| ctx_lh_postcentral | 58.08 | 0.64 | 0.57 |
| ctx_lh_posteriorcingulate | 59.03 | 0.51 | 0.38 |
| ctx_lh_precentral | 64.94 | 1.03 | 0.92 |
| ctx_lh_precuneus | 43.60 | 0.40 | 0.31 |
| ctx_lh_rostralanteriorcingulate | 50.90 | 1.46 | 0.85 |
| ctx_lh_rostralmiddlefrontal | 75.55 | 0.79 | 0.53 |
| ctx_lh_superiorfrontal | 62.59 | 1.24 | 0.85 |
| ctx_lh_superiorparietal | 42.11 | 0.72 | 0.48 |
| ctx_lh_superiortemporal | 68.13 | 1.67 | 0.97 |
| ctx_lh_supramarginal | 54.15 | 1.26 | 0.79 |
| ctx_lh_temporalpole | 70.44 | 3.94 | 1.14 |
| ctx_lh_transversetemporal | 58.75 | 1.10 | 0.66 |
| ctx_rh_caudalanteriorcingulate | 65.09 | 1.82 | 1.01 |
| ctx_rh_caudalmiddlefrontal | 123.80 | 1.14 | 0.66 |
| ctx_rh_cuneus | 41.44 | 0.46 | 0.43 |
| ctx_rh_entorhinal | 93.23 | 3.95 | 1.44 |
| ctx_rh_frontalpole | 86.32 | 0.92 | 0.37 |
| ctx_rh_fusiform | 72.01 | 1.70 | 1.20 |
| ctx_rh_inferiorparietal | 70.99 | 1.24 | 0.87 |
| ctx_rh_inferiortemporal | 99.68 | 2.00 | 1.08 |
| ctx_rh_insula | 106.64 | 2.80 | 1.42 |
| ctx_rh_isthmuscingulate | 59.94 | 0.03 | 0.02 |
| ctx_rh_lateraloccipital | 45.75 | 0.71 | 0.54 |
| ctx_rh_lateralorbitofrontal | 117.36 | 1.21 | 0.75 |
| ctx_rh_lingual | 42.77 | 0.52 | 0.49 |
| ctx_rh_medialorbitofrontal | 91.64 | 1.40 | 0.84 |
| ctx_rh_middletemporal | 135.67 | 1.63 | 0.97 |
| ctx_rh_paracentral | 71.29 | 1.10 | 0.81 |
| ctx_rh_parahippocampal | 73.25 | 2.16 | 1.15 |
| ctx_rh_parsopercularis | 134.62 | 1.27 | 0.83 |
| ctx_rh_parsorbitalis | 141.55 | 1.32 | 0.56 |
| ctx_rh_parstriangularis | 168.90 | 1.38 | 0.82 |
| ctx_rh_pericalcarine | 40.04 | 0.48 | 0.44 |
| ctx_rh_postcentral | 121.90 | 0.57 | 0.46 |
| ctx_rh_posteriorcingulate | 66.93 | 0.51 | 0.45 |
| ctx_rh_precentral | 124.62 | 1.06 | 0.76 |
| ctx_rh_precuneus | 52.72 | 0.40 | 0.46 |
| ctx_rh_rostralanteriorcingulate | 65.27 | 2.35 | 0.92 |
| ctx_rh_rostralmiddlefrontal | 110.98 | 0.99 | 0.52 |
| ctx_rh_superiorfrontal | 79.41 | 1.29 | 0.80 |
| ctx_rh_superiorparietal | 55.95 | 0.66 | 0.51 |
| ctx_rh_superiortemporal | 129.79 | 2.09 | 1.11 |
| ctx_rh_supramarginal | 109.40 | 1.24 | 0.70 |
| ctx_rh_temporalpole | 104.45 | 5.24 | 1.78 |
| ctx_rh_transversetemporal | 94.82 | 1.39 | 0.80 |

**3 A.)** Detailed results of the multivariate analysis of ΔVOL ~ EF + age + number of ECT in 85 regions.

(t_EF_: t value of EF, p_EF_: corresponding p value, t_age_: t value of age, p_age_: corresponding p value, t_nECT_: t value of number of ECT, p_nECT_: corresponding p value, F: F value of the model, p_FDR_: FDR corrected p value of the EF (α=0.05)):

| ROI | t_EF_ | p_EF_ | t_age_ | p_age_ | t_nECT_ | p_nECT_ | F | p_FDR_ |
| --- | --- | --- | --- | --- | --- | --- | --- | --- |
| Left_Cerebellum_Cortex | 0.6 | 0.56 | 1.4 | 0.18 | 0.8 | 0.41 | 0.8 | 0.82 |
| Left_Thalamus | 1.4 | 0.16 | 1.7 | 0.10 | 3.0 | 0.00 | 5.1 | 0.46 |
| Left_Caudate | 0.8 | 0.41 | 2.0 | 0.05 | 3.8 | 0.00 | 7.3 | 0.73 |
| Left_Putamen | 1.6 | 0.10 | 2.1 | 0.03 | 1.4 | 0.15 | 3.8 | 0.37 |
| Left_Pallidum | 1.3 | 0.20 | 3.1 | 0.00 | 3.4 | 0.00 | 8.8 | 0.46 |
| Brain_Stem | -0.3 | 0.78 | 1.0 | 0.33 | -0.2 | 0.82 | 0.5 | 0.94 |
| Left_Hippocampus | 7.0 | 0.00 | -1.9 | 0.05 | 5.4 | 0.00 | 42.8 | 0.00 |
| Left_Amygdala | 9.2 | 0.00 | 0.6 | 0.54 | 7.9 | 0.00 | 72.8 | 0.00 |
| Left_Accumbens_area | 3.2 | 0.00 | -1.4 | 0.16 | 3.1 | 0.00 | 11.2 | 0.02 |
| Left_VentralDC | 0.3 | 0.74 | 0.8 | 0.43 | 3.2 | 0.00 | 3.8 | 0.93 |
| Right_Cerebellum_Cortex | 0.8 | 0.44 | 1.8 | 0.08 | 0.8 | 0.44 | 1.2 | 0.74 |
| Right_Thalamus | 0.1 | 0.91 | 1.7 | 0.08 | 3.3 | 0.00 | 4.6 | 0.96 |
| Right_Caudate | 0.2 | 0.86 | 3.7 | 0.00 | 2.7 | 0.01 | 6.8 | 0.94 |
| Right_Putamen | 0.0 | 0.99 | 1.6 | 0.11 | 2.5 | 0.01 | 2.9 | 0.99 |
| Right_Pallidum | 0.1 | 0.95 | 3.6 | 0.00 | 3.1 | 0.00 | 7.2 | 0.97 |
| Right_Hippocampus | 2.1 | 0.03 | -0.1 | 0.89 | 6.0 | 0.00 | 16.7 | 0.16 |
| Right_Amygdala | -0.2 | 0.80 | 3.6 | 0.00 | 10.5 | 0.00 | 42.4 | 0.94 |
| Right_Accumbens_area | 1.3 | 0.19 | 0.6 | 0.57 | 4.6 | 0.00 | 8.4 | 0.46 |
| Right_VentralDC | -0.0 | 0.96 | 1.3 | 0.20 | 1.7 | 0.10 | 1.4 | 0.97 |
| ctx_lh_caudalanteriorcingulate | 1.0 | 0.33 | 3.3 | 0.00 | 3.9 | 0.00 | 8.1 | 0.65 |
| ctx_lh_caudalmiddlefrontal | -2.0 | 0.04 | 2.9 | 0.00 | 2.4 | 0.02 | 6.0 | 0.18 |
| ctx_lh_cuneus | 3.2 | 0.00 | 0.2 | 0.86 | 1.9 | 0.07 | 4.1 | 0.02 |
| ctx_lh_entorhinal | 4.8 | 0.00 | -0.4 | 0.67 | 4.9 | 0.00 | 23.4 | 0.00 |
| ctx_lh_fusiform | 2.1 | 0.03 | 2.1 | 0.03 | 4.0 | 0.00 | 10.6 | 0.16 |
| ctx_lh_inferiorparietal | 0.5 | 0.60 | 4.1 | 0.00 | 2.8 | 0.01 | 8.5 | 0.87 |
| ctx_lh_inferiortemporal | 1.5 | 0.14 | 2.9 | 0.00 | 3.1 | 0.00 | 8.0 | 0.43 |
| ctx_lh_isthmuscingulate | 1.6 | 0.12 | 0.2 | 0.85 | 1.3 | 0.19 | 1.2 | 0.40 |
| ctx_lh_lateraloccipital | 0.4 | 0.71 | 1.7 | 0.09 | 2.3 | 0.02 | 2.6 | 0.93 |
| ctx_lh_lateralorbitofrontal | 2.3 | 0.02 | 0.3 | 0.77 | 2.9 | 0.00 | 6.8 | 0.12 |
| ctx_lh_lingual | 1.2 | 0.23 | 1.3 | 0.20 | 1.9 | 0.05 | 1.8 | 0.49 |
| ctx_lh_medialorbitofrontal | 2.4 | 0.02 | 1.1 | 0.28 | 4.2 | 0.00 | 10.9 | 0.12 |
| ctx_lh_middletemporal | 1.3 | 0.19 | 2.6 | 0.01 | 4.0 | 0.00 | 9.8 | 0.46 |
| ctx_lh_parahippocampal | 1.3 | 0.18 | -0.0 | 1.00 | 3.7 | 0.00 | 6.9 | 0.46 |
| ctx_lh_paracentral | 0.7 | 0.48 | 1.5 | 0.12 | 2.3 | 0.02 | 2.3 | 0.77 |
| ctx_lh_parsopercularis | -0.7 | 0.47 | 2.7 | 0.01 | 2.6 | 0.01 | 4.6 | 0.77 |
| ctx_lh_parsorbitalis | 2.0 | 0.05 | -0.4 | 0.67 | 2.2 | 0.03 | 4.3 | 0.20 |
| ctx_lh_parstriangularis | 0.7 | 0.46 | 1.8 | 0.07 | 3.9 | 0.00 | 7.1 | 0.77 |
| ctx_lh_pericalcarine | 2.6 | 0.01 | 1.6 | 0.10 | 2.7 | 0.01 | 4.2 | 0.07 |
| ctx_lh_postcentral | -0.5 | 0.65 | -1.0 | 0.32 | 2.0 | 0.05 | 1.7 | 0.90 |
| ctx_lh_posteriorcingulate | 2.5 | 0.01 | 3.2 | 0.00 | 1.8 | 0.07 | 4.6 | 0.10 |
| ctx_lh_precentral | -1.5 | 0.12 | 2.0 | 0.05 | 3.0 | 0.00 | 4.6 | 0.40 |
| ctx_lh_precuneus | 2.8 | 0.01 | 2.4 | 0.02 | 1.3 | 0.21 | 3.4 | 0.06 |
| ctx_lh_rostralanteriorcingulate | -0.2 | 0.86 | 0.9 | 0.37 | 3.7 | 0.00 | 4.8 | 0.94 |
| ctx_lh_rostralmiddlefrontal | 0.9 | 0.38 | -0.2 | 0.87 | 3.0 | 0.00 | 4.3 | 0.70 |
| ctx_lh_superiorfrontal | -1.2 | 0.22 | 2.2 | 0.03 | 2.5 | 0.01 | 4.7 | 0.01 |
| ctx_lh_superiorparietal | 0.3 | 0.73 | 3.1 | 0.00 | 1.4 | 0.15 | 3.9 | 0.15 |
| ctx_lh_superiortemporal | 3.0 | 0.00 | 0.1 | 0.94 | 4.3 | 0.00 | 13.6 | 0.00 |
| ctx_lh_supramarginal | -1.2 | 0.23 | 2.2 | 0.03 | 3.0 | 0.00 | 5.1 | 0.00 |
| ctx_lh_frontalpole | 0.2 | 0.88 | 0.5 | 0.61 | 2.3 | 0.02 | 2.1 | 0.02 |
| ctx_lh_temporalpole | 4.6 | 0.00 | 2.4 | 0.02 | 4.8 | 0.00 | 23.2 | 0.00 |
| ctx_lh_transversetemporal | 0.8 | 0.43 | 0.6 | 0.57 | 3.4 | 0.00 | 5.2 | 0.00 |
| ctx_lh_insula | 1.8 | 0.08 | 1.3 | 0.19 | 4.4 | 0.00 | 10.8 | 0.00 |
| ctx_rh_caudalanteriorcingulate | 1.4 | 0.17 | 4.6 | 0.00 | 4.1 | 0.00 | 11.6 | 0.00 |
| ctx_rh_caudalmiddlefrontal | 0.1 | 0.91 | 5.2 | 0.00 | 0.6 | 0.55 | 9.1 | 0.55 |
| ctx_rh_cuneus | 1.4 | 0.17 | 2.2 | 0.03 | 1.7 | 0.08 | 2.4 | 0.08 |
| ctx_rh_entorhinal | 0.7 | 0.50 | 1.9 | 0.06 | 5.5 | 0.00 | 12.2 | 0.00 |
| ctx_rh_fusiform | 0.3 | 0.75 | 3.7 | 0.00 | 3.9 | 0.00 | 9.2 | 0.00 |
| ctx_rh_inferiorparietal | -0.9 | 0.34 | 5.7 | 0.00 | 1.0 | 0.33 | 13.7 | 0.33 |
| ctx_rh_inferiortemporal | 0.3 | 0.74 | 4.7 | 0.00 | 3.3 | 0.00 | 10.5 | 0.00 |
| ctx_rh_isthmuscingulate | 0.2 | 0.83 | 0.4 | 0.69 | 0.3 | 0.73 | 0.1 | 0.73 |
| ctx_rh_lateraloccipital | 1.4 | 0.17 | 2.8 | 0.01 | 1.9 | 0.05 | 3.5 | 0.05 |
| ctx_rh_lateralorbitofrontal | 1.1 | 0.27 | 1.3 | 0.20 | 2.4 | 0.02 | 3.3 | 0.02 |
| ctx_rh_lingual | 0.4 | 0.71 | 1.1 | 0.25 | 2.0 | 0.04 | 1.7 | 0.04 |
| ctx_rh_medialorbitofrontal | 1.4 | 0.17 | 2.8 | 0.01 | 3.6 | 0.00 | 8.2 | 0.00 |
| ctx_rh_middletemporal | 0.1 | 0.96 | 5.0 | 0.00 | 2.7 | 0.01 | 11.1 | 0.01 |
| ctx_rh_parahippocampal | -0.6 | 0.55 | 0.8 | 0.42 | 3.9 | 0.00 | 5.3 | 0.00 |
| ctx_rh_paracentral | -0.3 | 0.74 | 2.8 | 0.00 | 1.4 | 0.17 | 3.5 | 0.17 |
| ctx_rh_parsopercularis | -1.7 | 0.08 | 4.3 | 0.00 | 2.0 | 0.05 | 10.4 | 0.05 |
| ctx_rh_parsorbitalis | 0.2 | 0.88 | 0.7 | 0.51 | 2.2 | 0.03 | 1.8 | 0.03 |
| ctx_rh_parstriangularis | -2.1 | 0.04 | 3.3 | 0.00 | 1.9 | 0.05 | 8.8 | 0.05 |
| ctx_rh_pericalcarine | 2.2 | 0.03 | 2.5 | 0.01 | 2.9 | 0.00 | 4.7 | 0.00 |
| ctx_rh_postcentral | 1.3 | 0.20 | 2.9 | 0.00 | 1.4 | 0.15 | 3.4 | 0.15 |
| ctx_rh_posteriorcingulate | 2.2 | 0.03 | 3.7 | 0.00 | 0.3 | 0.80 | 5.0 | 0.80 |
| ctx_rh_precentral | 0.5 | 0.65 | 4.1 | 0.00 | 1.9 | 0.06 | 6.5 | 0.06 |
| ctx_rh_precuneus | 1.0 | 0.30 | 3.5 | 0.00 | 0.1 | 0.92 | 4.2 | 0.92 |
| ctx_rh_rostralanteriorcingulate | 3.2 | 0.00 | 3.5 | 0.00 | 5.6 | 0.00 | 16.4 | 0.00 |
| ctx_rh_rostralmiddlefrontal | -0.9 | 0.39 | 1.8 | 0.07 | 1.5 | 0.14 | 2.4 | 0.14 |
| ctx_rh_superiorfrontal | 0.3 | 0.79 | 3.9 | 0.00 | 2.2 | 0.03 | 6.4 | 0.03 |
| ctx_rh_superiorparietal | 0.2 | 0.82 | 3.6 | 0.00 | 0.4 | 0.72 | 4.4 | 0.72 |
| ctx_rh_superiortemporal | 2.4 | 0.02 | 4.4 | 0.00 | 3.9 | 0.00 | 11.3 | 0.00 |
| ctx_rh_supramarginal | 0.6 | 0.53 | 6.4 | 0.00 | 1.1 | 0.25 | 14.6 | 0.25 |
| ctx_rh_frontalpole | 0.2 | 0.85 | 0.4 | 0.66 | 2.1 | 0.03 | 1.8 | 0.03 |
| ctx_rh_temporalpole | -0.2 | 0.82 | 3.9 | 0.00 | 7.3 | 0.00 | 22.2 | 0.00 |
| ctx_rh_transversetemporal | 0.7 | 0.49 | 4.4 | 0.00 | 2.2 | 0.03 | 8.0 | 0.03 |
| ctx_rh_insula | -1.0 | 0.34 | 4.1 | 0.00 | 5.7 | 0.00 | 16.4 | 0.00 |

**3 B.)** Detailed results of the multivariate analysis of ΔVOL ~ EF + age + number of ECT in 85 regions without the N=151 subjects from Argyelan et al. 2019, showing that these results are not only driven by those individuals.

(t_EF_: t value of EF, p_EF_: corresponding p value, t_age_: t value of age, p_age_: corresponding p value, t_nECT_: t value of number of ECT, p_nECT_: corresponding p value, F: F value of the model, p_FDR_: FDR corrected p value of the EF (α=0.05)):

| ROI | t_EF_ | p_EF_ | t_age_ | p_age_ | t_nECT_ | p_nECT_ | F | p_FDR_ |
| --- | --- | --- | --- | --- | --- | --- | --- | --- |
| Left_Cerebellum_Cortex | 1.0 | 0.31 | 2.1 | 0.03 | 0.1 | 0.96 | 1.6 | 0.55 |
| Left_Thalamus | 1.4 | 0.18 | 1.6 | 0.12 | 2.3 | 0.02 | 3.6 | 0.42 |
| Left_Caudate | 0.6 | 0.58 | 2.6 | 0.01 | 3.5 | 0.00 | 6.9 | 0.78 |
| Left_Putamen | 1.6 | 0.11 | 2.6 | 0.01 | 1.4 | 0.16 | 4.7 | 0.41 |
| Left_Pallidum | 1.2 | 0.22 | 3.2 | 0.00 | 3.2 | 0.00 | 8.5 | 0.49 |
| Brain_Stem | -0.5 | 0.64 | 0.9 | 0.36 | -0.3 | 0.78 | 0.5 | 0.80 |
| Left_Hippocampus | 4.7 | 0.00 | -0.3 | 0.75 | 4.8 | 0.00 | 19.8 | 0.00 |
| Left_Amygdala | 7.5 | 0.00 | 0.2 | 0.83 | 6.9 | 0.00 | 45.2 | 0.00 |
| Left_Accumbens_area | 3.7 | 0.00 | -0.3 | 0.73 | 2.5 | 0.01 | 8.7 | 0.01 |
| Left_VentralDC | -0.3 | 0.75 | 0.8 | 0.42 | 3.0 | 0.00 | 3.3 | 0.87 |
| Right_Cerebellum_Cortex | 0.1 | 0.89 | 2.2 | 0.03 | 0.4 | 0.71 | 1.7 | 0.93 |
| Right_Thalamus | 0.7 | 0.49 | 1.0 | 0.31 | 2.5 | 0.01 | 2.7 | 0.75 |
| Right_Caudate | 0.7 | 0.49 | 3.9 | 0.00 | 1.8 | 0.07 | 6.8 | 0.75 |
| Right_Putamen | -0.6 | 0.58 | 2.2 | 0.03 | 2.5 | 0.01 | 3.6 | 0.78 |
| Right_Pallidum | 1.3 | 0.20 | 2.8 | 0.01 | 2.6 | 0.01 | 6.0 | 0.47 |
| Right_Hippocampus | 1.5 | 0.13 | 0.0 | 0.97 | 5.7 | 0.00 | 13.5 | 0.41 |
| Right_Amygdala | -0.4 | 0.67 | 2.7 | 0.01 | 9.5 | 0.00 | 33.1 | 0.81 |
| Right_Accumbens_area | 1.4 | 0.16 | 0.5 | 0.65 | 3.7 | 0.00 | 5.9 | 0.41 |
| Right_VentralDC | 0.4 | 0.72 | 1.5 | 0.14 | 1.4 | 0.16 | 1.4 | 0.86 |
| ctx_lh_caudalanteriorcingulate | 1.5 | 0.12 | 2.8 | 0.00 | 3.1 | 0.00 | 5.8 | 0.41 |
| ctx_lh_caudalmiddlefrontal | -1.0 | 0.30 | 2.9 | 0.00 | 1.3 | 0.20 | 3.6 | 0.55 |
| ctx_lh_cuneus | 2.8 | 0.01 | -0.6 | 0.53 | 1.3 | 0.19 | 4.0 | 0.06 |
| ctx_lh_entorhinal | 3.8 | 0.00 | -0.4 | 0.68 | 4.3 | 0.00 | 13.9 | 0.00 |
| ctx_lh_fusiform | 1.6 | 0.12 | 1.4 | 0.17 | 3.6 | 0.00 | 7.1 | 0.41 |
| ctx_lh_inferiorparietal | 0.6 | 0.57 | 3.3 | 0.00 | 2.2 | 0.03 | 5.6 | 0.78 |
| ctx_lh_inferiortemporal | 1.4 | 0.17 | 2.8 | 0.01 | 2.8 | 0.01 | 7.0 | 0.41 |
| ctx_lh_isthmuscingulate | 1.7 | 0.09 | 0.4 | 0.71 | 0.5 | 0.62 | 1.1 | 0.37 |
| ctx_lh_lateraloccipital | 0.8 | 0.44 | 1.2 | 0.23 | 2.2 | 0.03 | 2.1 | 0.71 |
| ctx_lh_lateralorbitofrontal | 2.0 | 0.04 | 0.2 | 0.82 | 2.7 | 0.01 | 4.8 | 0.29 |
| ctx_lh_lingual | 1.1 | 0.29 | 1.1 | 0.28 | 1.2 | 0.22 | 1.1 | 0.54 |
| ctx_lh_medialorbitofrontal | 1.8 | 0.08 | 1.5 | 0.15 | 3.9 | 0.00 | 8.0 | 0.37 |
| ctx_lh_middletemporal | 1.2 | 0.23 | 2.9 | 0.00 | 3.5 | 0.00 | 8.7 | 0.49 |
| ctx_lh_parahippocampal | 0.2 | 0.82 | -1.0 | 0.29 | 3.2 | 0.00 | 4.1 | 0.91 |
| ctx_lh_paracentral | 1.5 | 0.13 | 1.4 | 0.17 | 1.1 | 0.28 | 1.4 | 0.41 |
| ctx_lh_parsopercularis | -0.5 | 0.63 | 2.1 | 0.03 | 1.7 | 0.09 | 2.4 | 0.80 |
| ctx_lh_parsorbitalis | 1.7 | 0.08 | -0.4 | 0.71 | 1.7 | 0.08 | 2.6 | 0.37 |
| ctx_lh_parstriangularis | 1.0 | 0.32 | 1.7 | 0.10 | 3.0 | 0.00 | 4.9 | 0.55 |
| ctx_lh_pericalcarine | 1.9 | 0.06 | 1.6 | 0.11 | 2.6 | 0.01 | 3.6 | 0.33 |
| ctx_lh_postcentral | -2.0 | 0.05 | -0.4 | 0.73 | 1.8 | 0.07 | 1.9 | 0.29 |
| ctx_lh_posteriorcingulate | 2.8 | 0.01 | 2.9 | 0.00 | 0.8 | 0.40 | 4.0 | 0.06 |
| ctx_lh_precentral | -1.0 | 0.31 | 2.0 | 0.04 | 1.9 | 0.06 | 2.8 | 0.55 |
| ctx_lh_precuneus | 3.2 | 0.00 | 1.9 | 0.06 | 0.4 | 0.71 | 3.6 | 0.02 |
| ctx_lh_rostralanteriorcingulate | 0.3 | 0.75 | 1.9 | 0.06 | 3.1 | 0.00 | 4.5 | 0.87 |
| ctx_lh_rostralmiddlefrontal | 1.1 | 0.28 | -0.0 | 0.99 | 2.4 | 0.02 | 2.9 | 0.54 |
| ctx_lh_superiorfrontal | 0.1 | 0.96 | 2.2 | 0.03 | 1.7 | 0.10 | 2.7 | 0.97 |
| ctx_lh_superiorparietal | 1.2 | 0.25 | 2.6 | 0.01 | 0.5 | 0.60 | 2.5 | 0.51 |
| ctx_lh_superiortemporal | 2.4 | 0.02 | 0.5 | 0.65 | 3.8 | 0.00 | 8.7 | 0.14 |
| ctx_lh_supramarginal | -1.6 | 0.11 | 2.3 | 0.02 | 2.7 | 0.01 | 4.9 | 0.41 |
| ctx_lh_frontalpole | 0.5 | 0.59 | 0.8 | 0.40 | 2.4 | 0.02 | 2.5 | 0.79 |
| ctx_lh_temporalpole | 4.1 | 0.00 | 2.2 | 0.03 | 4.4 | 0.00 | 17.6 | 0.00 |
| ctx_lh_transversetemporal | 0.5 | 0.65 | 0.4 | 0.66 | 2.4 | 0.02 | 2.3 | 0.80 |
| ctx_lh_insula | 1.1 | 0.28 | 1.5 | 0.12 | 3.5 | 0.00 | 6.2 | 0.54 |
| ctx_rh_caudalanteriorcingulate | 1.4 | 0.15 | 3.1 | 0.00 | 3.5 | 0.00 | 7.1 | 0.41 |
| ctx_rh_caudalmiddlefrontal | 1.2 | 0.23 | 4.8 | 0.00 | -0.1 | 0.92 | 7.6 | 0.49 |
| ctx_rh_cuneus | 1.5 | 0.15 | 1.5 | 0.12 | 1.5 | 0.15 | 1.7 | 0.41 |
| ctx_rh_entorhinal | -0.8 | 0.44 | 1.4 | 0.16 | 5.2 | 0.00 | 9.8 | 0.71 |
| ctx_rh_fusiform | 0.5 | 0.63 | 2.8 | 0.01 | 3.0 | 0.00 | 5.7 | 0.80 |
| ctx_rh_inferiorparietal | -1.0 | 0.33 | 4.2 | 0.00 | 1.2 | 0.22 | 8.6 | 0.57 |
| ctx_rh_inferiortemporal | 0.6 | 0.56 | 3.4 | 0.00 | 2.5 | 0.01 | 6.1 | 0.78 |
| ctx_rh_isthmuscingulate | 0.0 | 0.99 | -0.3 | 0.79 | 0.2 | 0.82 | 0.0 | 0.99 |
| ctx_rh_lateraloccipital | 0.5 | 0.63 | 1.5 | 0.14 | 2.1 | 0.04 | 2.1 | 0.80 |
| ctx_rh_lateralorbitofrontal | 0.6 | 0.54 | 1.5 | 0.15 | 2.0 | 0.05 | 2.3 | 0.78 |
| ctx_rh_lingual | 0.6 | 0.54 | 1.1 | 0.29 | 1.5 | 0.14 | 1.1 | 0.78 |
| ctx_rh_medialorbitofrontal | 0.2 | 0.85 | 2.0 | 0.04 | 3.3 | 0.00 | 5.3 | 0.91 |
| ctx_rh_middletemporal | 0.1 | 0.94 | 3.3 | 0.00 | 2.0 | 0.05 | 5.4 | 0.97 |
| ctx_rh_parahippocampal | -1.4 | 0.17 | -0.3 | 0.73 | 3.1 | 0.00 | 3.7 | 0.41 |
| ctx_rh_paracentral | 0.3 | 0.80 | 1.6 | 0.11 | 1.0 | 0.34 | 1.1 | 0.89 |
| ctx_rh_parsopercularis | -0.3 | 0.79 | 3.9 | 0.00 | 0.7 | 0.50 | 6.2 | 0.89 |
| ctx_rh_parsorbitalis | -0.2 | 0.84 | 1.2 | 0.23 | 1.5 | 0.14 | 1.3 | 0.91 |
| ctx_rh_parstriangularis | -1.7 | 0.09 | 3.3 | 0.00 | 1.2 | 0.24 | 7.2 | 0.37 |
| ctx_rh_pericalcarine | 1.5 | 0.14 | 2.5 | 0.01 | 2.8 | 0.01 | 4.6 | 0.41 |
| ctx_rh_postcentral | 1.6 | 0.12 | 2.9 | 0.00 | 0.7 | 0.46 | 3.2 | 0.41 |
| ctx_rh_posteriorcingulate | 2.3 | 0.02 | 2.2 | 0.03 | 0.0 | 0.98 | 2.4 | 0.19 |
| ctx_rh_precentral | 1.2 | 0.24 | 4.1 | 0.00 | 0.8 | 0.43 | 5.9 | 0.50 |
| ctx_rh_precuneus | 1.5 | 0.15 | 2.7 | 0.01 | -0.3 | 0.79 | 2.5 | 0.41 |
| ctx_rh_rostralanteriorcingulate | 3.1 | 0.00 | 2.7 | 0.01 | 4.6 | 0.00 | 12.5 | 0.03 |
| ctx_rh_rostralmiddlefrontal | -0.1 | 0.96 | 2.2 | 0.03 | 0.6 | 0.56 | 1.9 | 0.97 |
| ctx_rh_superiorfrontal | 1.7 | 0.09 | 3.7 | 0.00 | 1.0 | 0.30 | 5.2 | 0.37 |
| ctx_rh_superiorparietal | 0.9 | 0.36 | 3.6 | 0.00 | -0.2 | 0.86 | 4.2 | 0.61 |
| ctx_rh_superiortemporal | 2.2 | 0.03 | 2.9 | 0.00 | 3.1 | 0.00 | 6.7 | 0.20 |
| ctx_rh_supramarginal | 1.4 | 0.17 | 5.4 | 0.00 | 0.8 | 0.44 | 9.8 | 0.41 |
| ctx_rh_frontalpole | 0.2 | 0.87 | -0.1 | 0.95 | 1.5 | 0.14 | 0.8 | 0.92 |
| ctx_rh_temporalpole | 0.4 | 0.73 | 4.1 | 0.00 | 6.4 | 0.00 | 19.7 | 0.86 |
| ctx_rh_transversetemporal | 0.7 | 0.46 | 3.0 | 0.00 | 1.5 | 0.13 | 3.8 | 0.72 |
| ctx_rh_insula | -0.6 | 0.52 | 3.0 | 0.00 | 4.8 | 0.00 | 11.2 | 0.78 |

Supplementary Table 4.

ΔMADRS ~ PC1_ΔVOL_ + PC2_ΔVOL_ + age + nECT (N=386, F_4,381_ = 15.95, p = 4x10^-12^)

|  | β | t | p |
| --- | --- | --- | --- |
| PC1_ΔVOL_ | -0.0015 | -0.51 | 0.61 |
| PC2 _ΔVOL_ | -0.016 | -2.35 | 0.019 |
| age | -0.0064 | -5.83 | <0.0001 |
| nECT | 0.0095 | 2.96 | 0.003 |

5 A) Loadings of the principal components of the PCA in the RUL patients. The "x", "y', "z" variables are the center coordinates of the regions in "ROI". The other columns contain the loadings in the first two PCs respectively.

| x | y | z | EF-  PC1 | EF-  PC2 | VOL-  PC1 | VOL-  PC2 | ROI |
| --- | --- | --- | --- | --- | --- | --- | --- |
| -23.43 | -61.51 | -36.62 | 0.10 | -0.17 | 0.08 | 0.04 | Left-Cerebellum-Cortex |
| -11.24 | -19.25 | 6.40 | 0.12 | -0.06 | 0.13 | -0.02 | Left-Thalamus |
| -13.30 | 6.51 | 10.56 | 0.11 | -0.05 | 0.11 | -0.07 | Left-Caudate |
| -25.71 | 0.29 | -0.36 | 0.12 | -0.07 | 0.08 | 0.01 | Left-Putamen |
| -19.65 | -4.65 | -1.00 | 0.12 | -0.07 | 0.07 | 0.00 | Left-Pallidum |
| 0.54 | -30.39 | -32.71 | 0.12 | -0.05 | 0.07 | 0.05 | Brain-Stem |
| -25.28 | -23.00 | -14.03 | 0.12 | -0.09 | 0.08 | -0.19 | Left-Hippocampus |
| -23.08 | -4.98 | -19.68 | 0.12 | -0.05 | 0.09 | -0.18 | Left-Amygdala |
| -8.87 | 11.96 | -6.61 | 0.12 | -0.09 | 0.08 | -0.19 | Left-Accumbens-area |
| -11.04 | -15.31 | -9.91 | 0.12 | -0.06 | 0.08 | 0.02 | Left-VentralDC |
| 23.93 | -61.09 | -36.84 | 0.11 | -0.11 | 0.06 | 0.10 | Right-Cerebellum-Cortex |
| 12.07 | -17.57 | 6.56 | 0.12 | 0.07 | 0.13 | -0.02 | Right-Thalamus |
| 13.73 | 8.88 | 10.08 | 0.09 | 0.20 | 0.09 | -0.07 | Right-Caudate |
| 26.38 | 2.46 | -1.41 | 0.09 | 0.25 | 0.09 | 0.03 | Right-Putamen |
| 20.73 | -3.57 | -1.09 | 0.10 | 0.19 | 0.07 | 0.01 | Right-Pallidum |
| 26.70 | -21.41 | -14.23 | 0.12 | 0.11 | 0.06 | -0.01 | Right-Hippocampus |
| 24.01 | -3.73 | -19.84 | 0.10 | 0.23 | 0.09 | -0.10 | Right-Amygdala |
| 9.30 | 12.10 | -7.09 | 0.12 | 0.04 | 0.09 | -0.14 | Right-Accumbens-area |
| 12.10 | -14.27 | -9.75 | 0.12 | 0.05 | 0.08 | 0.03 | Right-VentralDC |
| -5.63 | 17.27 | 27.94 | 0.12 | 0.06 | 0.13 | -0.13 | ctx-lh-caudalanteriorcingulate |
| -36.01 | 12.14 | 47.96 | 0.12 | -0.00 | 0.13 | 0.07 | ctx-lh-caudalmiddlefrontal |
| -4.69 | -82.92 | 17.45 | 0.11 | -0.11 | 0.11 | 0.06 | ctx-lh-cuneus |
| -22.78 | -4.88 | -34.44 | 0.11 | -0.06 | 0.07 | -0.22 | ctx-lh-entorhinal |
| -35.16 | -41.89 | -22.17 | 0.11 | -0.15 | 0.12 | -0.11 | ctx-lh-fusiform |
| -41.19 | -68.87 | 35.42 | 0.11 | -0.13 | 0.13 | 0.10 | ctx-lh-inferiorparietal |
| -51.44 | -35.26 | -21.73 | 0.11 | -0.16 | 0.12 | -0.04 | ctx-lh-inferiortemporal |
| -7.13 | -45.68 | 18.31 | 0.12 | -0.07 | 0.12 | -0.06 | ctx-lh-isthmuscingulate |
| -31.24 | -87.83 | 0.06 | 0.11 | -0.16 | 0.10 | 0.12 | ctx-lh-lateraloccipital |
| -23.88 | 30.63 | -17.65 | 0.11 | -0.00 | 0.10 | -0.15 | ctx-lh-lateralorbitofrontal |
| -11.22 | -71.21 | -5.00 | 0.11 | -0.15 | 0.11 | 0.02 | ctx-lh-lingual |
| -6.93 | 33.22 | -16.03 | 0.12 | 0.02 | 0.09 | -0.21 | ctx-lh-medialorbitofrontal |
| -58.89 | -23.92 | -12.76 | 0.11 | -0.15 | 0.11 | 0.00 | ctx-lh-middletemporal |
| -22.56 | -33.26 | -15.13 | 0.12 | -0.11 | 0.10 | -0.17 | ctx-lh-parahippocampal |
| -6.38 | -28.43 | 58.38 | 0.11 | 0.07 | 0.12 | 0.09 | ctx-lh-paracentral |
| -47.87 | 18.01 | 13.88 | 0.12 | -0.08 | 0.14 | 0.01 | ctx-lh-parsopercularis |
| -42.12 | 38.13 | -13.54 | 0.11 | -0.03 | 0.09 | -0.08 | ctx-lh-parsorbitalis |
| -46.47 | 35.29 | 1.70 | 0.12 | -0.06 | 0.12 | -0.03 | ctx-lh-parstriangularis |
| -10.06 | -81.43 | 5.09 | 0.11 | -0.14 | 0.09 | 0.12 | ctx-lh-pericalcarine |
| -43.69 | -24.25 | 47.47 | 0.08 | -0.01 | 0.11 | 0.06 | ctx-lh-postcentral |
| -6.12 | -19.27 | 35.79 | 0.12 | 0.05 | 0.13 | -0.02 | ctx-lh-posteriorcingulate |
| -40.17 | -6.96 | 43.17 | 0.12 | -0.00 | 0.14 | 0.08 | ctx-lh-precentral |
| -7.97 | -58.73 | 37.17 | 0.12 | -0.04 | 0.14 | 0.06 | ctx-lh-precuneus |
| -5.78 | 35.80 | 2.14 | 0.12 | 0.01 | 0.10 | -0.19 | ctx-lh-rostralanteriorcingulate |
| -31.14 | 47.93 | 18.15 | 0.12 | -0.01 | 0.11 | 0.01 | ctx-lh-rostralmiddlefrontal |
| -9.71 | 27.45 | 44.40 | 0.12 | 0.06 | 0.14 | 0.04 | ctx-lh-superiorfrontal |
| -21.09 | -66.77 | 49.28 | 0.11 | -0.01 | 0.12 | 0.14 | ctx-lh-superiorparietal |
| -52.84 | -10.36 | -4.42 | 0.12 | -0.13 | 0.12 | -0.09 | ctx-lh-superiortemporal |
| -54.10 | -36.45 | 32.44 | 0.12 | -0.11 | 0.13 | 0.05 | ctx-lh-supramarginal |
| -6.91 | 66.07 | -8.23 | 0.11 | 0.04 | 0.06 | -0.09 | ctx-lh-frontalpole |
| -28.80 | 12.38 | -37.90 | 0.06 | -0.04 | 0.08 | -0.15 | ctx-lh-temporalpole |
| -43.27 | -22.72 | 7.54 | 0.11 | -0.11 | 0.12 | -0.04 | ctx-lh-transversetemporal |
| -35.35 | -0.08 | -1.34 | 0.12 | -0.08 | 0.13 | -0.15 | ctx-lh-insula |
| 6.30 | 20.59 | 26.37 | 0.12 | 0.08 | 0.11 | 0.01 | ctx-rh-caudalanteriorcingulate |
| 38.02 | 11.21 | 48.79 | 0.07 | 0.15 | 0.12 | 0.15 | ctx-rh-caudalmiddlefrontal |
| 7.12 | -80.32 | 18.18 | 0.11 | -0.10 | 0.11 | 0.13 | ctx-rh-cuneus |
| 22.90 | -3.78 | -33.65 | 0.10 | 0.10 | 0.08 | -0.16 | ctx-rh-entorhinal |
| 35.74 | -38.34 | -24.19 | 0.12 | 0.05 | 0.13 | -0.03 | ctx-rh-fusiform |
| 47.45 | -60.76 | 33.97 | 0.10 | -0.06 | 0.11 | 0.21 | ctx-rh-inferiorparietal |
| 51.43 | -29.15 | -24.81 | 0.10 | 0.12 | 0.12 | 0.02 | ctx-rh-inferiortemporal |
| 8.72 | -44.82 | 16.50 | 0.12 | -0.05 | 0.11 | -0.06 | ctx-rh-isthmuscingulate |
| 34.82 | -83.93 | 1.86 | 0.11 | -0.12 | 0.10 | 0.17 | ctx-rh-lateraloccipital |
| 22.93 | 31.82 | -18.38 | 0.11 | 0.17 | 0.10 | -0.15 | ctx-rh-lateralorbitofrontal |
| 14.42 | -68.01 | -5.38 | 0.12 | -0.11 | 0.13 | 0.02 | ctx-rh-lingual |
| 6.27 | 37.72 | -16.65 | 0.12 | 0.02 | 0.11 | -0.19 | ctx-rh-medialorbitofrontal |
| 59.21 | -22.58 | -13.29 | 0.08 | 0.02 | 0.12 | 0.04 | ctx-rh-middletemporal |
| 22.94 | -29.88 | -16.82 | 0.12 | 0.03 | 0.11 | -0.13 | ctx-rh-parahippocampal |
| 7.23 | -24.08 | 56.41 | 0.09 | 0.11 | 0.13 | 0.11 | ctx-rh-paracentral |
| 48.75 | 15.61 | 11.22 | 0.09 | 0.25 | 0.13 | 0.07 | ctx-rh-parsopercularis |
| 44.12 | 39.79 | -12.04 | 0.07 | 0.11 | 0.08 | -0.08 | ctx-rh-parsorbitalis |
| 48.83 | 33.63 | 6.14 | 0.08 | 0.13 | 0.12 | 0.05 | ctx-rh-parstriangularis |
| 12.12 | -79.35 | 6.30 | 0.11 | -0.12 | 0.09 | 0.15 | ctx-rh-pericalcarine |
| 46.69 | -18.63 | 42.47 | 0.06 | 0.08 | 0.11 | 0.18 | ctx-rh-postcentral |
| 6.82 | -18.79 | 37.51 | 0.12 | 0.06 | 0.12 | 0.02 | ctx-rh-posteriorcingulate |
| 40.43 | -6.49 | 45.07 | 0.06 | 0.11 | 0.13 | 0.16 | ctx-rh-precentral |
| 10.02 | -57.15 | 38.02 | 0.11 | -0.02 | 0.13 | 0.11 | ctx-rh-precuneus |
| 7.06 | 36.51 | 5.73 | 0.12 | 0.03 | 0.11 | -0.14 | ctx-rh-rostralanteriorcingulate |
| 34.19 | 46.69 | 17.65 | 0.11 | 0.17 | 0.12 | 0.08 | ctx-rh-rostralmiddlefrontal |
| 12.22 | 30.75 | 44.21 | 0.10 | 0.14 | 0.13 | 0.09 | ctx-rh-superiorfrontal |
| 24.19 | -61.64 | 53.05 | 0.07 | 0.05 | 0.11 | 0.22 | ctx-rh-superiorparietal |
| 54.56 | -6.55 | -5.17 | 0.11 | 0.15 | 0.14 | -0.01 | ctx-rh-superiortemporal |
| 56.64 | -28.29 | 31.82 | 0.11 | 0.03 | 0.13 | 0.14 | ctx-rh-supramarginal |
| 8.37 | 65.27 | -10.84 | 0.10 | 0.01 | 0.06 | -0.11 | ctx-rh-frontalpole |
| 30.30 | 15.60 | -35.57 | 0.08 | 0.14 | 0.08 | -0.09 | ctx-rh-temporalpole |
| 45.30 | -19.09 | 7.74 | 0.11 | 0.16 | 0.12 | 0.03 | ctx-rh-transversetemporal |
| 36.85 | 1.53 | -1.80 | 0.10 | 0.22 | 0.13 | -0.05 | ctx-rh-insula |

5 B) Loadings of the principal components of the PCA in the BT patients. The "x", "y', "z" variables are the center coordinates of the regions in "ROI". The other columns contain the loadings in the first two PCs, respectively.

| x | y | z | EF-  PC1 | EF-  PC2 | VOL-  PC1 | VOL-  PC2 | ROI |
| --- | --- | --- | --- | --- | --- | --- | --- |
| -23.43 | -61.51 | -36.62 | 0.10 | -0.09 | 0.06 | 0.05 | Left-Cerebellum-Cortex |
| -11.24 | -19.25 | 6.40 | 0.13 | 0.03 | 0.10 | -0.04 | Left-Thalamus |
| -13.30 | 6.51 | 10.56 | 0.12 | 0.04 | 0.07 | -0.01 | Left-Caudate |
| -25.71 | 0.29 | -0.36 | 0.12 | 0.09 | 0.09 | 0.11 | Left-Putamen |
| -19.65 | -4.65 | -1.00 | 0.12 | 0.10 | 0.07 | -0.08 | Left-Pallidum |
| 0.54 | -30.39 | -32.71 | 0.12 | -0.00 | 0.03 | 0.15 | Brain-Stem |
| -25.28 | -23.00 | -14.03 | 0.13 | 0.05 | 0.09 | -0.20 | Left-Hippocampus |
| -23.08 | -4.98 | -19.68 | 0.12 | 0.09 | 0.10 | -0.23 | Left-Amygdala |
| -8.87 | 11.96 | -6.61 | 0.12 | 0.07 | 0.08 | -0.09 | Left-Accumbens-area |
| -11.04 | -15.31 | -9.91 | 0.13 | 0.06 | 0.07 | -0.08 | Left-VentralDC |
| 23.93 | -61.09 | -36.84 | 0.11 | -0.07 | 0.06 | 0.13 | Right-Cerebellum-Cortex |
| 12.07 | -17.57 | 6.56 | 0.12 | 0.04 | 0.11 | 0.03 | Right-Thalamus |
| 13.73 | 8.88 | 10.08 | 0.11 | 0.08 | 0.10 | 0.10 | Right-Caudate |
| 26.38 | 2.46 | -1.41 | 0.12 | 0.10 | 0.06 | 0.09 | Right-Putamen |
| 20.73 | -3.57 | -1.09 | 0.12 | 0.11 | 0.04 | -0.01 | Right-Pallidum |
| 26.70 | -21.41 | -14.23 | 0.13 | 0.05 | 0.04 | -0.16 | Right-Hippocampus |
| 24.01 | -3.73 | -19.84 | 0.13 | 0.10 | 0.07 | -0.24 | Right-Amygdala |
| 9.30 | 12.10 | -7.09 | 0.13 | 0.07 | 0.08 | -0.13 | Right-Accumbens-area |
| 12.10 | -14.27 | -9.75 | 0.13 | 0.06 | 0.06 | -0.05 | Right-VentralDC |
| -5.63 | 17.27 | 27.94 | 0.12 | -0.02 | 0.12 | -0.07 | ctx-lh-caudalanteriorcingulate |
| -36.01 | 12.14 | 47.96 | 0.10 | -0.03 | 0.12 | 0.08 | ctx-lh-caudalmiddlefrontal |
| -4.69 | -82.92 | 17.45 | 0.08 | -0.22 | 0.11 | 0.04 | ctx-lh-cuneus |
| -22.78 | -4.88 | -34.44 | 0.12 | 0.04 | 0.08 | -0.24 | ctx-lh-entorhinal |
| -35.16 | -41.89 | -22.17 | 0.12 | -0.05 | 0.12 | -0.14 | ctx-lh-fusiform |
| -41.19 | -68.87 | 35.42 | 0.09 | -0.19 | 0.12 | -0.01 | ctx-lh-inferiorparietal |
| -51.44 | -35.26 | -21.73 | 0.12 | 0.02 | 0.11 | -0.14 | ctx-lh-inferiortemporal |
| -7.13 | -45.68 | 18.31 | 0.10 | -0.13 | 0.10 | 0.07 | ctx-lh-isthmuscingulate |
| -31.24 | -87.83 | 0.06 | 0.08 | -0.20 | 0.11 | 0.02 | ctx-lh-lateraloccipital |
| -23.88 | 30.63 | -17.65 | 0.13 | 0.08 | 0.11 | -0.01 | ctx-lh-lateralorbitofrontal |
| -11.22 | -71.21 | -5.00 | 0.09 | -0.16 | 0.11 | -0.00 | ctx-lh-lingual |
| -6.93 | 33.22 | -16.03 | 0.12 | 0.01 | 0.09 | -0.07 | ctx-lh-medialorbitofrontal |
| -58.89 | -23.92 | -12.76 | 0.12 | 0.09 | 0.11 | -0.08 | ctx-lh-middletemporal |
| -22.56 | -33.26 | -15.13 | 0.12 | -0.01 | 0.10 | -0.19 | ctx-lh-parahippocampal |
| -6.38 | -28.43 | 58.38 | 0.10 | -0.15 | 0.11 | 0.04 | ctx-lh-paracentral |
| -47.87 | 18.01 | 13.88 | 0.11 | 0.14 | 0.14 | 0.05 | ctx-lh-parsopercularis |
| -42.12 | 38.13 | -13.54 | 0.12 | 0.11 | 0.11 | 0.01 | ctx-lh-parsorbitalis |
| -46.47 | 35.29 | 1.70 | 0.11 | 0.16 | 0.13 | 0.02 | ctx-lh-parstriangularis |
| -10.06 | -81.43 | 5.09 | 0.08 | -0.20 | 0.06 | 0.00 | ctx-lh-pericalcarine |
| -43.69 | -24.25 | 47.47 | 0.11 | 0.00 | 0.11 | 0.08 | ctx-lh-postcentral |
| -6.12 | -19.27 | 35.79 | 0.11 | -0.06 | 0.12 | 0.05 | ctx-lh-posteriorcingulate |
| -40.17 | -6.96 | 43.17 | 0.12 | 0.03 | 0.14 | 0.07 | ctx-lh-precentral |
| -7.97 | -58.73 | 37.17 | 0.09 | -0.19 | 0.11 | 0.03 | ctx-lh-precuneus |
| -5.78 | 35.80 | 2.14 | 0.12 | 0.02 | 0.08 | -0.04 | ctx-lh-rostralanteriorcingulate |
| -31.14 | 47.93 | 18.15 | 0.12 | 0.05 | 0.13 | 0.03 | ctx-lh-rostralmiddlefrontal |
| -9.71 | 27.45 | 44.40 | 0.11 | -0.08 | 0.14 | 0.02 | ctx-lh-superiorfrontal |
| -21.09 | -66.77 | 49.28 | 0.09 | -0.21 | 0.12 | 0.05 | ctx-lh-superiorparietal |
| -52.84 | -10.36 | -4.42 | 0.12 | 0.13 | 0.14 | -0.11 | ctx-lh-superiortemporal |
| -54.10 | -36.45 | 32.44 | 0.11 | -0.04 | 0.13 | 0.00 | ctx-lh-supramarginal |
| -6.91 | 66.07 | -8.23 | 0.11 | -0.04 | 0.10 | -0.02 | ctx-lh-frontalpole |
| -28.80 | 12.38 | -37.90 | 0.11 | 0.06 | 0.11 | -0.17 | ctx-lh-temporalpole |
| -43.27 | -22.72 | 7.54 | 0.12 | 0.08 | 0.11 | 0.03 | ctx-lh-transversetemporal |
| -35.35 | -0.08 | -1.34 | 0.12 | 0.13 | 0.14 | -0.12 | ctx-lh-insula |
| 6.30 | 20.59 | 26.37 | 0.12 | 0.00 | 0.12 | -0.12 | ctx-rh-caudalanteriorcingulate |
| 38.02 | 11.21 | 48.79 | 0.08 | -0.01 | 0.13 | 0.15 | ctx-rh-caudalmiddlefrontal |
| 7.12 | -80.32 | 18.18 | 0.07 | -0.24 | 0.12 | 0.14 | ctx-rh-cuneus |
| 22.90 | -3.78 | -33.65 | 0.12 | 0.02 | 0.05 | -0.23 | ctx-rh-entorhinal |
| 35.74 | -38.34 | -24.19 | 0.12 | -0.07 | 0.12 | -0.10 | ctx-rh-fusiform |
| 47.45 | -60.76 | 33.97 | 0.06 | -0.11 | 0.13 | 0.14 | ctx-rh-inferiorparietal |
| 51.43 | -29.15 | -24.81 | 0.12 | 0.00 | 0.11 | -0.05 | ctx-rh-inferiortemporal |
| 8.72 | -44.82 | 16.50 | 0.10 | -0.13 | 0.11 | 0.08 | ctx-rh-isthmuscingulate |
| 34.82 | -83.93 | 1.86 | 0.07 | -0.21 | 0.12 | 0.09 | ctx-rh-lateraloccipital |
| 22.93 | 31.82 | -18.38 | 0.12 | 0.09 | 0.09 | -0.01 | ctx-rh-lateralorbitofrontal |
| 14.42 | -68.01 | -5.38 | 0.09 | -0.20 | 0.12 | 0.10 | ctx-rh-lingual |
| 6.27 | 37.72 | -16.65 | 0.12 | 0.02 | 0.11 | -0.14 | ctx-rh-medialorbitofrontal |
| 59.21 | -22.58 | -13.29 | 0.05 | 0.04 | 0.10 | -0.02 | ctx-rh-middletemporal |
| 22.94 | -29.88 | -16.82 | 0.12 | -0.03 | 0.10 | -0.16 | ctx-rh-parahippocampal |
| 7.23 | -24.08 | 56.41 | 0.09 | -0.15 | 0.12 | 0.08 | ctx-rh-paracentral |
| 48.75 | 15.61 | 11.22 | 0.10 | 0.12 | 0.13 | 0.10 | ctx-rh-parsopercularis |
| 44.12 | 39.79 | -12.04 | 0.09 | 0.10 | 0.11 | 0.11 | ctx-rh-parsorbitalis |
| 48.83 | 33.63 | 6.14 | 0.08 | 0.10 | 0.13 | 0.07 | ctx-rh-parstriangularis |
| 12.12 | -79.35 | 6.30 | 0.07 | -0.24 | 0.10 | 0.10 | ctx-rh-pericalcarine |
| 46.69 | -18.63 | 42.47 | 0.09 | 0.02 | 0.12 | 0.10 | ctx-rh-postcentral |
| 6.82 | -18.79 | 37.51 | 0.11 | -0.08 | 0.12 | 0.04 | ctx-rh-posteriorcingulate |
| 40.43 | -6.49 | 45.07 | 0.09 | 0.05 | 0.14 | 0.14 | ctx-rh-precentral |
| 10.02 | -57.15 | 38.02 | 0.09 | -0.22 | 0.12 | 0.16 | ctx-rh-precuneus |
| 7.06 | 36.51 | 5.73 | 0.12 | 0.02 | 0.09 | -0.20 | ctx-rh-rostralanteriorcingulate |
| 34.19 | 46.69 | 17.65 | 0.08 | 0.03 | 0.14 | 0.11 | ctx-rh-rostralmiddlefrontal |
| 12.22 | 30.75 | 44.21 | 0.11 | -0.06 | 0.14 | 0.07 | ctx-rh-superiorfrontal |
| 24.19 | -61.64 | 53.05 | 0.08 | -0.20 | 0.12 | 0.14 | ctx-rh-superiorparietal |
| 54.56 | -6.55 | -5.17 | 0.09 | 0.11 | 0.13 | -0.06 | ctx-rh-superiortemporal |
| 56.64 | -28.29 | 31.82 | 0.07 | -0.02 | 0.13 | 0.14 | ctx-rh-supramarginal |
| 8.37 | 65.27 | -10.84 | 0.11 | -0.05 | 0.10 | -0.03 | ctx-rh-frontalpole |
| 30.30 | 15.60 | -35.57 | 0.12 | 0.07 | 0.09 | -0.17 | ctx-rh-temporalpole |
| 45.30 | -19.09 | 7.74 | 0.12 | 0.09 | 0.12 | 0.08 | ctx-rh-transversetemporal |
| 36.85 | 1.53 | -1.80 | 0.12 | 0.13 | 0.14 | -0.11 | ctx-rh-insula |

5 C) Loadings of the principal components of the PCA in the MIX patients. The "x", "y', "z" variables are the center coordinates of the regions in "ROI". The other columns contain the loadings in the first two PCs, respectively.

| x | y | z | EF-  PC1 | EF-  PC2 | VOL-  PC1 | VOL-  PC2 | ROI |
| --- | --- | --- | --- | --- | --- | --- | --- |
| -23.43 | -61.51 | -36.62 | 0.10 | -0.05 | 0.11 | -0.08 | Left-Cerebellum-Cortex |
| -11.24 | -19.25 | 6.40 | 0.14 | 0.01 | 0.08 | -0.18 | Left-Thalamus |
| -13.30 | 6.51 | 10.56 | 0.11 | 0.12 | 0.10 | -0.07 | Left-Caudate |
| -25.71 | 0.29 | -0.36 | 0.10 | 0.15 | 0.08 | -0.16 | Left-Putamen |
| -19.65 | -4.65 | -1.00 | 0.11 | 0.14 | 0.08 | -0.16 | Left-Pallidum |
| 0.54 | -30.39 | -32.71 | 0.13 | -0.01 | 0.05 | -0.09 | Brain-Stem |
| -25.28 | -23.00 | -14.03 | 0.12 | 0.11 | 0.02 | -0.23 | Left-Hippocampus |
| -23.08 | -4.98 | -19.68 | 0.11 | 0.14 | 0.04 | -0.22 | Left-Amygdala |
| -8.87 | 11.96 | -6.61 | 0.13 | 0.06 | 0.11 | -0.01 | Left-Accumbens-area |
| -11.04 | -15.31 | -9.91 | 0.14 | -0.01 | 0.05 | -0.13 | Left-VentralDC |
| 23.93 | -61.09 | -36.84 | 0.10 | -0.07 | 0.11 | -0.11 | Right-Cerebellum-Cortex |
| 12.07 | -17.57 | 6.56 | 0.13 | 0.00 | 0.10 | -0.14 | Right-Thalamus |
| 13.73 | 8.88 | 10.08 | 0.11 | 0.06 | 0.12 | -0.04 | Right-Caudate |
| 26.38 | 2.46 | -1.41 | 0.11 | 0.06 | 0.10 | -0.09 | Right-Putamen |
| 20.73 | -3.57 | -1.09 | 0.12 | 0.06 | 0.07 | -0.18 | Right-Pallidum |
| 26.70 | -21.41 | -14.23 | 0.13 | 0.02 | 0.00 | -0.20 | Right-Hippocampus |
| 24.01 | -3.73 | -19.84 | 0.12 | 0.09 | 0.09 | -0.15 | Right-Amygdala |
| 9.30 | 12.10 | -7.09 | 0.13 | -0.00 | 0.09 | -0.11 | Right-Accumbens-area |
| 12.10 | -14.27 | -9.75 | 0.14 | -0.03 | 0.03 | -0.19 | Right-VentralDC |
| -5.63 | 17.27 | 27.94 | 0.10 | -0.15 | 0.13 | -0.00 | ctx-lh-caudalanteriorcingulate |
| -36.01 | 12.14 | 47.96 | 0.13 | 0.06 | 0.12 | 0.08 | ctx-lh-caudalmiddlefrontal |
| -4.69 | -82.92 | 17.45 | 0.06 | -0.19 | 0.11 | 0.08 | ctx-lh-cuneus |
| -22.78 | -4.88 | -34.44 | 0.09 | 0.16 | 0.05 | -0.22 | ctx-lh-entorhinal |
| -35.16 | -41.89 | -22.17 | 0.11 | 0.10 | 0.10 | -0.21 | ctx-lh-fusiform |
| -41.19 | -68.87 | 35.42 | 0.12 | -0.09 | 0.13 | 0.06 | ctx-lh-inferiorparietal |
| -51.44 | -35.26 | -21.73 | 0.09 | 0.14 | 0.12 | -0.10 | ctx-lh-inferiortemporal |
| -7.13 | -45.68 | 18.31 | 0.09 | -0.17 | 0.13 | 0.09 | ctx-lh-isthmuscingulate |
| -31.24 | -87.83 | 0.06 | 0.10 | -0.13 | 0.09 | -0.07 | ctx-lh-lateraloccipital |
| -23.88 | 30.63 | -17.65 | 0.09 | 0.17 | 0.11 | 0.01 | ctx-lh-lateralorbitofrontal |
| -11.22 | -71.21 | -5.00 | 0.10 | -0.11 | 0.10 | -0.10 | ctx-lh-lingual |
| -6.93 | 33.22 | -16.03 | 0.11 | 0.11 | 0.10 | -0.02 | ctx-lh-medialorbitofrontal |
| -58.89 | -23.92 | -12.76 | 0.10 | 0.15 | 0.12 | -0.05 | ctx-lh-middletemporal |
| -22.56 | -33.26 | -15.13 | 0.12 | 0.06 | 0.09 | -0.06 | ctx-lh-parahippocampal |
| -6.38 | -28.43 | 58.38 | 0.09 | -0.15 | 0.11 | 0.10 | ctx-lh-paracentral |
| -47.87 | 18.01 | 13.88 | 0.11 | 0.08 | 0.13 | 0.08 | ctx-lh-parsopercularis |
| -42.12 | 38.13 | -13.54 | 0.09 | 0.15 | 0.09 | 0.05 | ctx-lh-parsorbitalis |
| -46.47 | 35.29 | 1.70 | 0.11 | 0.12 | 0.11 | 0.06 | ctx-lh-parstriangularis |
| -10.06 | -81.43 | 5.09 | 0.08 | -0.17 | 0.09 | 0.04 | ctx-lh-pericalcarine |
| -43.69 | -24.25 | 47.47 | 0.11 | 0.04 | 0.12 | 0.11 | ctx-lh-postcentral |
| -6.12 | -19.27 | 35.79 | 0.08 | -0.18 | 0.14 | 0.05 | ctx-lh-posteriorcingulate |
| -40.17 | -6.96 | 43.17 | 0.13 | 0.03 | 0.13 | 0.09 | ctx-lh-precentral |
| -7.97 | -58.73 | 37.17 | 0.08 | -0.18 | 0.12 | 0.11 | ctx-lh-precuneus |
| -5.78 | 35.80 | 2.14 | 0.11 | -0.06 | 0.12 | -0.00 | ctx-lh-rostralanteriorcingulate |
| -31.14 | 47.93 | 18.15 | 0.11 | 0.12 | 0.12 | 0.09 | ctx-lh-rostralmiddlefrontal |
| -9.71 | 27.45 | 44.40 | 0.13 | -0.05 | 0.13 | 0.11 | ctx-lh-superiorfrontal |
| -21.09 | -66.77 | 49.28 | 0.11 | -0.10 | 0.13 | 0.09 | ctx-lh-superiorparietal |
| -52.84 | -10.36 | -4.42 | 0.11 | 0.13 | 0.12 | -0.09 | ctx-lh-superiortemporal |
| -54.10 | -36.45 | 32.44 | 0.13 | 0.01 | 0.12 | 0.07 | ctx-lh-supramarginal |
| -6.91 | 66.07 | -8.23 | 0.09 | 0.13 | 0.09 | 0.09 | ctx-lh-frontalpole |
| -28.80 | 12.38 | -37.90 | 0.08 | 0.16 | 0.06 | -0.16 | ctx-lh-temporalpole |
| -43.27 | -22.72 | 7.54 | 0.12 | 0.09 | 0.09 | -0.10 | ctx-lh-transversetemporal |
| -35.35 | -0.08 | -1.34 | 0.10 | 0.15 | 0.13 | -0.07 | ctx-lh-insula |
| 6.30 | 20.59 | 26.37 | 0.11 | -0.13 | 0.13 | 0.04 | ctx-rh-caudalanteriorcingulate |
| 38.02 | 11.21 | 48.79 | 0.10 | -0.10 | 0.12 | 0.18 | ctx-rh-caudalmiddlefrontal |
| 7.12 | -80.32 | 18.18 | 0.07 | -0.18 | 0.11 | 0.02 | ctx-rh-cuneus |
| 22.90 | -3.78 | -33.65 | 0.12 | 0.08 | 0.07 | -0.16 | ctx-rh-entorhinal |
| 35.74 | -38.34 | -24.19 | 0.13 | 0.01 | 0.13 | -0.15 | ctx-rh-fusiform |
| 47.45 | -60.76 | 33.97 | 0.09 | -0.15 | 0.14 | 0.01 | ctx-rh-inferiorparietal |
| 51.43 | -29.15 | -24.81 | 0.11 | 0.04 | 0.13 | -0.10 | ctx-rh-inferiortemporal |
| 8.72 | -44.82 | 16.50 | 0.09 | -0.16 | 0.10 | 0.08 | ctx-rh-isthmuscingulate |
| 34.82 | -83.93 | 1.86 | 0.09 | -0.16 | 0.10 | -0.04 | ctx-rh-lateraloccipital |
| 22.93 | 31.82 | -18.38 | 0.12 | 0.09 | 0.13 | 0.05 | ctx-rh-lateralorbitofrontal |
| 14.42 | -68.01 | -5.38 | 0.11 | -0.12 | 0.10 | -0.09 | ctx-rh-lingual |
| 6.27 | 37.72 | -16.65 | 0.13 | 0.04 | 0.11 | -0.01 | ctx-rh-medialorbitofrontal |
| 59.21 | -22.58 | -13.29 | 0.08 | 0.02 | 0.13 | -0.02 | ctx-rh-middletemporal |
| 22.94 | -29.88 | -16.82 | 0.13 | -0.03 | 0.10 | -0.13 | ctx-rh-parahippocampal |
| 7.23 | -24.08 | 56.41 | 0.09 | -0.13 | 0.10 | 0.06 | ctx-rh-paracentral |
| 48.75 | 15.61 | 11.22 | 0.12 | -0.02 | 0.14 | 0.13 | ctx-rh-parsopercularis |
| 44.12 | 39.79 | -12.04 | 0.11 | 0.03 | 0.11 | 0.05 | ctx-rh-parsorbitalis |
| 48.83 | 33.63 | 6.14 | 0.10 | 0.01 | 0.13 | 0.07 | ctx-rh-parstriangularis |
| 12.12 | -79.35 | 6.30 | 0.08 | -0.16 | 0.09 | -0.01 | ctx-rh-pericalcarine |
| 46.69 | -18.63 | 42.47 | 0.08 | -0.11 | 0.13 | 0.13 | ctx-rh-postcentral |
| 6.82 | -18.79 | 37.51 | 0.09 | -0.17 | 0.12 | 0.05 | ctx-rh-posteriorcingulate |
| 40.43 | -6.49 | 45.07 | 0.08 | -0.11 | 0.13 | 0.12 | ctx-rh-precentral |
| 10.02 | -57.15 | 38.02 | 0.08 | -0.17 | 0.11 | 0.09 | ctx-rh-precuneus |
| 7.06 | 36.51 | 5.73 | 0.12 | -0.03 | 0.13 | 0.02 | ctx-rh-rostralanteriorcingulate |
| 34.19 | 46.69 | 17.65 | 0.13 | -0.01 | 0.13 | 0.16 | ctx-rh-rostralmiddlefrontal |
| 12.22 | 30.75 | 44.21 | 0.12 | -0.09 | 0.12 | 0.16 | ctx-rh-superiorfrontal |
| 24.19 | -61.64 | 53.05 | 0.09 | -0.13 | 0.13 | 0.08 | ctx-rh-superiorparietal |
| 54.56 | -6.55 | -5.17 | 0.13 | -0.00 | 0.13 | -0.02 | ctx-rh-superiortemporal |
| 56.64 | -28.29 | 31.82 | 0.10 | -0.14 | 0.14 | 0.10 | ctx-rh-supramarginal |
| 8.37 | 65.27 | -10.84 | 0.11 | 0.07 | 0.08 | 0.02 | ctx-rh-frontalpole |
| 30.30 | 15.60 | -35.57 | 0.11 | 0.08 | 0.09 | -0.03 | ctx-rh-temporalpole |
| 45.30 | -19.09 | 7.74 | 0.12 | -0.04 | 0.08 | -0.14 | ctx-rh-transversetemporal |
| 36.85 | 1.53 | -1.80 | 0.12 | 0.03 | 0.14 | -0.06 | ctx-rh-insula |

Supplementary Table 6. A

ΔMADRS ~ PC1_EF_ + PC2_EF_ + age + nECT (N=386, F_4,381_ = 15.55, p = 9x10^-12^)

|  | β | t | p |
| --- | --- | --- | --- |
| PC1_EF_ | 0.005 | 2.11 | 0.036 |
| PC2 _EF_ | -0.001 | -0.19 | 0.85 |
| age | -0.0059 | -4.97 | <0.0001 |
| nECT | 0.01 | 3.21 | 0.001 |

Supplementary Table 6. B

ΔMADRS ~ PC1_EF_ + PC2_ΔVOL_ + age + nECT (N=386, F_4,381_ = 16.66, p = 1x10^-12^)

|  | β | t | p |
| --- | --- | --- | --- |
| PC1_EF_ | 0.0041 | 1.64 | 0.1 |
| PC2 _ΔVOL_ | -0.014 | -1.96 | 0.05 |
| age | -0.0057 | -4.97 | <0.0001 |
| nECT | 0.009 | 2.76 | 0.006 |
